# Three new *Leptographium* spp. (Ophiostomatales) infecting hardwood trees in Norway and Poland

**DOI:** 10.1007/s10482-018-1123-8

**Published:** 2018-07-06

**Authors:** Robert Jankowiak, Agnieszka Ostafińska, Truls Aas, Halvor Solheim, Piotr Bilański, Riikka Linnakoski, Georg Hausner

**Affiliations:** 1 Department of Forest Pathology, Mycology and Tree Physiology, Institute of Forest Ecosystem Protection, University of Agriculture in Krakow, Al. 29 Listopada 46, 31-425 Kraków, Poland; 2 Faculty of Environmental Sciences and Natural Resource Management, Norwegian University of Life Sciences, P.O. Box 5003, 1432 Ås, Norway; 3 Norwegian Institute of Bioeconomy Research, P.O. Box 115, 1431 Ås, Norway; 4 Department of Forest Protection, Entomology and Forest Climatology, Institute of Forest Ecosystem Protection, University of Agriculture in Krakow, Al. 29 Listopada 46, 31-425 Kraków, Poland; 5 Natural Resources Institute Finland (Luke), Latokartanonkaari 9, 00790 Helsinki, Finland; 6 Department of Microbiology, Buller Building 213, University of Manitoba, Winnpeg, R3T 2N2 Canada

**Keywords:** Bark beetle-associated fungi, *Leptographium flavum*, *Leptographium tardum*, *Leptographium vulnerum*, Ophiostomatoid fungi, Phylogeny, Three novel species, Tree wound

## Abstract

Species of *Leptographium* are characterized by mononematous or synnematous conidiophores and are commonly associated with different arthropods. Some of them also produce a sexual state characterised by globose ascomata with elongated necks. Compared to investigations on coniferous trees, the occurrence of *Leptographium* species on hardwood trees has been poorly studied in Europe. During a survey of ophiostomatoid fungi on various hardwood tree species in Norway and Poland, three unusual species, which fit in the broader morphological description of *Leptographium* spp., were found in association with *Trypodendron domesticum*, *Trypodendron signatum* and *Dryocoetes alni*, and from wounds on a variety of hardwoods. Phylogenetic analyses of sequence data for six different loci (ITS1–5.8 S–ITS2, ITS2-LSU, ACT, β-tubulin, CAL, and TEF-1α) showed that these *Leptographium* species are phylogenetically closely related to the species of the *Grosmannia olivacea* complex. The first species forms a well-supported lineage that includes *Ophiostoma brevicolle*, while the two other new taxa resided in a separate lineage; possibly affiliated with *Grosmannia francke*-*grosmanniae*. All the new species produce perithecia with necks terminating in ostiolar hyphae and orange-section shaped ascospores with cucullate, gelatinous sheaths. These species also produce dark olivaceous mononematous asexual states in culture. In addition, two of the newly described species have a second type of conidiophore with a short and non-pigmented stipe. The new *Leptographium* species can be easily distinguished from each other by their appearance and growth in culture. Based on novel morphological characters and distinct DNA sequences, these fungi were recognised as new taxa for which the names *Leptographium tardum* sp. nov., *Leptographium vulnerum* sp. nov., and *Leptographium flavum* sp. nov. are provided.

**Electronic supplementary material:**

The online version of this article (10.1007/s10482-018-1123-8) contains supplementary material, which is available to authorized users.

## Introduction

Species of *Leptographium* Lagerb. and Melin (Lagerberg et al. 1927) are commonly associated with bark beetles and weevils (Jacobs and Wingfield 2001). Most members are causal agents of blue stain of timber, having the potential to cause economic losses in the forestry industry (Jacobs et al. 2006). Some important tree diseases are also known, e.g. *Leptographium wageneri* complex species that is responsible for black stain root disease (BSRD) on conifers in western North America (Goheen and Cobb 1978).

In contrast to the conifers, occurrence of *Leptographium* spp. on hardwood trees has been relatively poorly investigated in Europe. *Leptographium* species have previously been isolated from the roots of various hardwood trees in the southeastern United States (Jacobs et al. 2006), and from beetles infesting hardwoods in China (Paciura et al. 2010). In Europe, *Grosmannia francke*-*grosmanniae* (R.W. Davidson) Zipfel, Z.W. de Beer and M.J. Wingf. was for a long time the only *Leptographium/Grosmannia* species reported from hardwoods (Davidson 1971; Jacobs and Wingfield 2001). Our recent research (Jankowiak et al. 2017) expanded the knowledge of hardwood-infecting *Leptographium* spp. in Europe by describing two new species of *Leptographium* that were assigned to the newly defined *Grosmannia grandifoliae* species complex.

In general, *Leptographium* spp. have mononematous, darkly pigmented conidiophores terminating in several series of branches giving rise to a brush-like conidiogenous structures that produce conidia in slimy masses, facilitating insect dispersal (Jacobs et al. 2001). However, species belonging to the *Grosmannia olivacea* complex form also synnematous conidiophores (De Beer and Wingfield 2013). In addition, some *Leptographium* species have a distinct well-developed sporothrix-like or hyalorhinocladiella-like synanamorphs (Jacobs and Wingfield 2001)*. Leptographium* spp. also produce sexual states that have historically been classified in various genera including *Grosmannia* Goid. (Goidànich 1936), *Ceratocystis* Ellis and Halst. (Upadhyay 1981), and *Ophiostoma* Syd. and P. Syd. (Seifert et al. 1993). Zipfel et al. (2006) based on phylogenies derived from ribosomal large subunit (LSU) and beta-tubulin sequences, distinguished between *Ophiostoma* and *Grosmannia*, and redefined the latter genus to include all *Leptographium* spp. with sexual states.

Following the “one fungus one name” principles adopted in the Melbourne Code (Hawksworth 2011; Taylor 2011), De Beer and Wingfield (2013) re-evaluated the taxonomy of *Leptographium* and *Grosmannia*, considering all available DNA sequence data for all species previously treated in either of the two genera. Ninety-four species were included and ten species complexes were defined within a broadly defined concept for *Leptographium* sensu lato, based on phylogenies resulting from analysing ribosomal internal transcribed spacer (ITS) and partial LSU sequences. The authors recognized that sequence data for additional gene regions would be necessary to fully resolve the delineation of *Leptographium* and *Grosmannia*, for which the type species *Leptographium lundbergii* and *Grosmannia penicillata* respectively, grouped in distinct species complexes. De Beer and Wingfield (2013) suggested that all known *Leptographium* and *Grosmannia* spp. placed in *Leptographium s. l.* based on phylogenetic inference, should be treated in their current genera (*Leptographium* or *Grosmannia*). However, new species, excluding those residing in the *G. penicillata* complex, should provisionally be treated in *Leptographium*, irrespective of their sexual or asexual morphs.

One of the species complexes recognized in *Leptographium s. l.* by De Beer and Wingfield (2013) was the *Grosmannia olivacea* complex. Earlier, Zipfel et al. (2006) transferred to *Grosmannia* spp., several species previously treated in the genus *Pesotum* (Okada et al. 1998) that also produced a sexual state. Massoumi Alamouti et al. (2007), Six et al. (2011) and Linnakoski et al. (2012) showed that additional species with synnematous asexual states also group within a monophyletic lineage along with *G. olivacea.* Currently, the *G. olivacea* complex is comprised of six species including *G. olivacea* (Math.-Käärik) Zipfel, Z.W. De Beer and M.J. Wingf., *G. sagmatospora* (E.F. Wright and Cain) Zipfel, Z.W. De Beer and M.J. Wingf., *G. olivaceapini* (R.W. Davidson) Z.W. de Beer, Linnak. and M.J. Wingf., *G. cucullata* (H. Solheim) Zipfel, Z.W. De Beer and M.J. Wingf., *G. davidsonii* (Olechow. and J. Reid) Zipfel, Z.W. De Beer and M.J. Wingf., and *G. vesca* (R.W. Davidson) Zipfel, Z.W. De Beer and M.J. Wingf. The status of three other species, *Graphium album* (Corda) Sacc., *G. francke*-*grosmanniae* (R.W. Davidson) Zipfel, Z.W. De Beer and M.J. Wingf. and *Ophiostoma brevicolle* (R.W. Davidson) de Hoog and R.J. Scheff. is unclear. Based on previously published sequences and morphology data, these probably also belong to the *G. olivacea* complex (De Beer and Wingfield 2013). The species residing in this complex are well-characterised by sharing morphologically similar sexual and asexual states. They produce globose ascomata with cylindrical necks, terminating in prominent ostiolar hyphae on which sticky droplets containing orange-section shaped ascospores with cucullate gelatinous sheaths are formed (Mathiesen 1951; Davidson 1958, 1971; Wright and Cain^1961^; Olchowecki and Reid 1974; Solheim 1986). The asexual morphs are more variable, including the synnematous and mononematous asexual states with conidiogenous cells producing conidia showing annellations.

During a survey of ophiostomatoid fungi on hardwoods in Poland and Norway, three undescribed *Leptographium* species with a sexual state resembling species in the *G. olivacea* complex were isolated from different bark and ambrosia beetle species, as well as from tree wounds. The aim of this study was to identify the undescribed fungi and provide evidence to designate them as new taxa within *Leptographium* by comparing their morphology and DNA sequences with that of known species.

## Materials and methods

### Isolations, fungal isolates and herbarium specimens

**Fig. 1:**
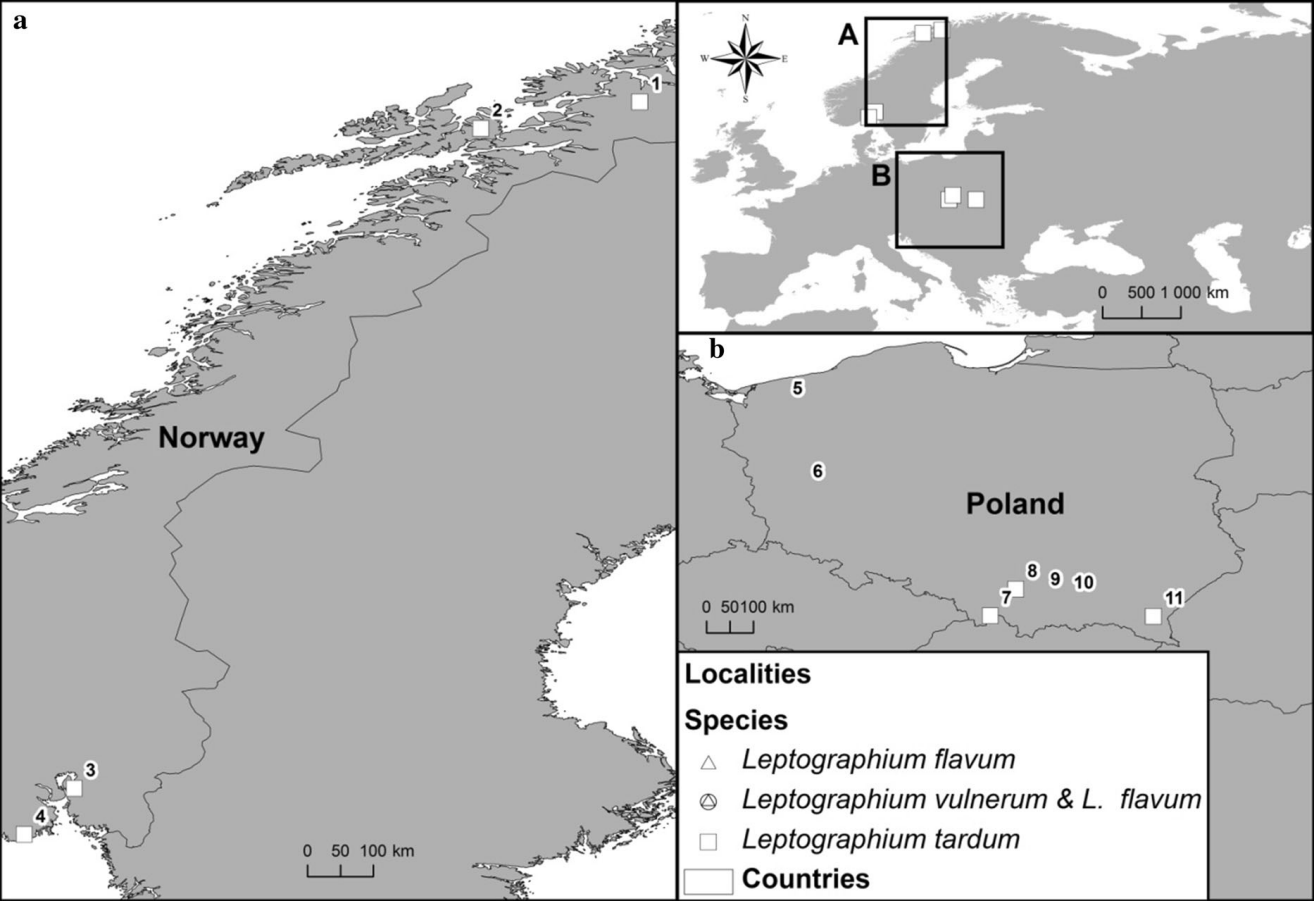
Origin of isolates used in this study: 1—Bardufoss, Målselv, Norway (69°4′5.54″N, 18°28′46.01″E); 2—Rudda, Kvæfjord, Norway (68°42′14.36″N, 16°18′33.53″E); 3—Syverud, Ås, Norway (59°41′20.79″N, 10°45′10.18″E); 4—Tagtvedt, Larvik, Norway (59°3′37.00″N 10°4′6.68″)E; 5—Resko, Poland (53°45′56.18″N, 15°25′19.25″E); 6—Babimost, Poland (52°10′23.42″N, 15°48′37.38″E); 7—Sopotnia, Poland (49°35′39.54″N, 19°16′39.02″E) 8—Zabierzów, Poland (50°6′26.01″N, 19°46′11.71″E); 9—Ispina, Poland (50°6′20.61″N, 20°22′11.85″E); 10—Wierzchosławice, Poland, 50°2′21.06″N, 20°48′49.32″E, 11—Rozpucie, Poland (49°34′59.71″N, 22°24′19.38″E)

**Fig. 2:**
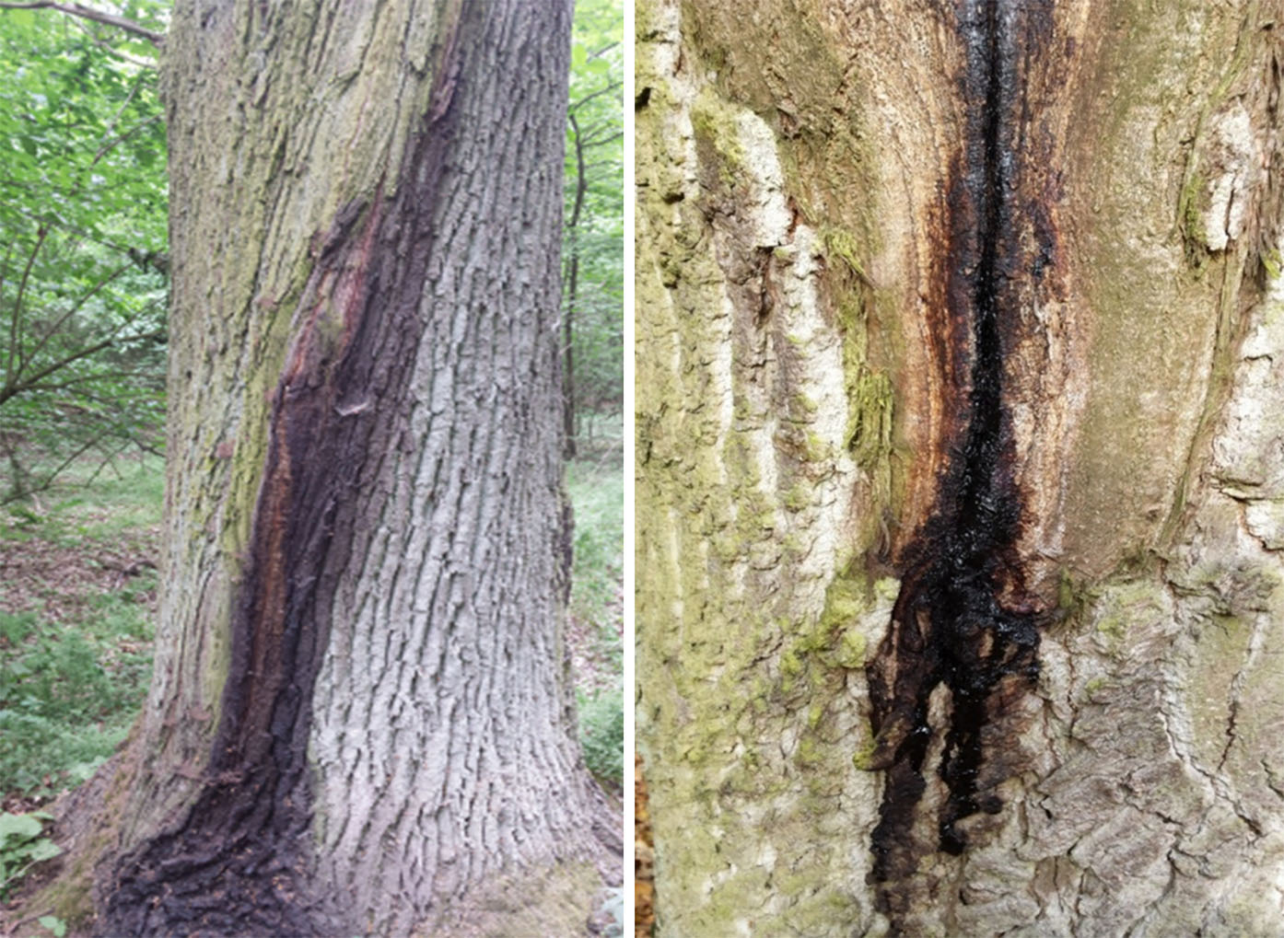
Active bleeding lesions on oak stems (Ispina study site)

Isolations were made from the beetles *Trypodendron domesticum* L., *T. signatum* Fabr. and *Dryocoetes alni* (Georg) in Norway, and *T. domesticum* and *Anisandrus dispar* (F.) in Poland. The adult beetles were excised from galleries established on decaying trees of *Alnus incana* (L.) (Norway), and on *Fagus sylvatica* L. and *Quercus robur* L. (Poland) with sterilised tweezers and stored individually in sterile 1.5 ml Eppendorf tubes for later isolations. Isolations were also performed on active bleeding lesions of *Carpinus betulus* L., *F. sylvatica* and *Q. robur* in Poland (Figs. 1, 2). These stem lesions were most likely caused by frost damage. Samples were collected at seven localities in Poland during January-October 2011–2016 and from four localities during September 2015–September 2016 in Norway (Fig. 1).

Fungal isolations were made directly from beetles by crushing them onto the surface of 2% Malt Extract Agar (MEA) (20 g Biocorp malt extract, 20 g agar, 1000 mL distilled water), containing cycloheximide (200 mg, Aldrich-Sigma, St. Louis, Co. LLC.) and tetracycline sulphate (200 mg, Polfa, Tarchomin SA). The agar plates were subsequently incubated at 22 °C for 7–14 days and later examined for fungal growth. In Norway, each bark beetle was divided into three parts, elytra, head and the rest, before placing the parts in three different Petri dishes containing 2% MEA without cycloheximide and tetracycline sulphate.

Fungal isolations were made from internal wood necrosis by sampling from cambium and wood within the range of the bleeding lesions up to a depth of 2 mm. First the inner bark was excised with a sterile axe and small portions of discoloured tissue (4 × 4 mm) were collected from the reaction zone of necrotised tissues with the aid of a sterile chisel, and plated on MEA with cycloheximide.

All fungal isolates used in this study are listed in Table 1. These isolates were maintained in the culture collection of the Department of Forest Pathology, Mycology and Tree Physiology; University of Agriculture in Krakow, Poland. The Norwegian isolates are stored at the culture collection of the Norwegian Institute of Bioeconomy. Ex-type isolates of new species described in this study were deposited in the Westerdijk Fungal Biodiversity Institute (CBS), Utrecht, the Netherlands, and in the culture collection (CMW) of the Forestry and Agricultural Biotechnology Institute (FABI), University of Pretoria, South Africa. Type specimens were deposited in the Herbarium of the University of Turku, Finland (TUR), Finland. A culture of *Ophiostoma brevicolle*, which is closely related to *Leptographium* taxon 1 was sourced from the culture collection of University of Manitoba as WIN(M)811 (= CBS150.78) in Canada (Table 1). Taxonomic descriptions and nomenclatural data were registered in MycoBank (www.MycoBank.org) (Robert et al. 2013).

**Table 1:** Isolates used in the present study

Species	Isolate no^1^				Host^2^	Insect^3^, substrate	Origin	GenBank accession no.					
	CMW	CBS	Herbarium	Other				ITS	ITS2-LSU	βT	TEF1-α	ACT	CAL
*L. tardum* sp. nov.	51782	144085		N2016-0625/1/1	*A. incana*	*T. domesticum*	Rudda, Norway	MH055522	MH055551	MH055579	MH055608	MH496042	MH496071
*L. tardum*	51783	144086		N2015-1552/3/10	*A. incana*	*D. alni*	Rudda, N	MH055521	MH055550	MH055578	MH055607	MH496041	MH496070
*L. tardum*	51784	144087^a^	http://mus.utu.fi/TFU.207249 ^P^	N2016-1614/3/1	*A. incana*	*T. signatum*	Bardufoss, N	MH055525	MH055554	MH055582	MH055611	MH496045	MH496073
*L. tardum*	51785	144088		N2016-1627/2/1	*A. incana*	*T. signatum*	Bardufoss, N	MH055526	MH055555	MH055583	MH055612	MH496046	
*L. tardum*				N2016-1631/1/2	*A. incana*	*T. signatum*	Bardufoss, N	MH055527	MH055556	MH055584	MH055613	MH496047	MH496074
*L. tardum*	51786	144089		N2016-0637/1/2	*A. incana*	*D. alni*	Syverud,N	MH055523	MH055552	MH055580	MH055609	MH496043	
*L. tardum*	51787			N2016-0676/2/2	*A. incana*	*T. domesticum*	Tagtvedt, N	MH055524	MH055553	MH055581	MH055610	MH496044	MH496072
*L. tardum*	51788	144090	http://mus.utu.fi/TFU.207250	KFL118TD	*F. sylvatica*	*T. domesticum*	Sopotnia, Poland	MH055528	MH055557	MH055585	MH055614	MH496048	MH496075
*L. tardum*	51789	144091^a^	http://mus.utu.fi/TFU.207251 ^H^	KFL29715TD	*F. sylvatica*	*T. domesticum*	Rozpucie, PL	MH055529	MH055558	MH055586	MH055615	MH496049	MH496076
*L. tardum*	51790	144092^a^	http://mus.utu.fi/TFU.207252 ^P^	KFL5814TD	*F. sylvatica*	*T. domesticum*	Zabierzów, PL	MH055530	MH055559	MH055587	MH055616	MH496050	MH496077
*L. tardum*	51791	144093	http://mus.utu.fi/TFU.207253	KFL6014TD	*F. sylvatica*	*T. domesticum*	Zabierzów, PL	MH055531	MH055560	MH055588	MH055617	MH496051	MH496078
*L. vulnerum* sp. nov.	51792	144094^a^	http://mus.utu.fi/TFU.207254 ^P^	KFL27716NGB	*C. betulus*	Tree wound	Babimost, PL	MH055536	MH055565	MH055593	MH055622	MH496056	MH496083
*L. vulnerum*	51793	144095		KFL27316NGB	*C. betulus*	Tree wound	Babimost, PL	MH055535	MH055564	MH055592	MH055621	MH496055	MH496082
*L. vulnerum*	51794	144096^a^	http://mus.utu.fi/TFU.207255 ^H^	KFL27216NBK	*F. sylvatica*	Tree wound	Babimost, PL	MH055534	MH055563	MH055591	MH055620	MH496054	MH496081
*L. vulnerum*	51795	144097^a^	http://mus.utu.fi/TFU.207256 ^P^	KFL110016NDBCZ	*Q. rubra*	Tree wound	Wierzchosławice, PL	MH055532	MH055561	MH055589	MH055618	MH496052	MH496079
*L. vulnerum*	51796	144098		KFL111416NDB	*Q. robur*	Tree wound	Wierzchosławice, PL	MH055533	MH055562	MH055590	MH055619	MH496053	MH496080
*L. flavum* sp. nov.	51797	144099	http://mus.utu.fi/TFU.207257 ^H^	KFL615NDB	*Q. robur*	Tree wound	Wierzchosławice, PL	MH055548	MH055577	MH055605	MH055634	MH496068	MH496095
*L. flavum*	51798	144100	http://mus.utu.fi/TFU.207258	KFL42016NDB	*Q. robur*	Tree wound	Ispina, PL	MH055547	MH055576	MH055604	MH055633	MH496067	MH496094
*L. flavum*	51799	144101	http://mus.utu.fi/TFU.207259	KFL41716NDB	*Q. robur*	Tree wound	Ispina, PL	MH055546	MH055575	MH055603	MH055632	MH496066	MH496093
*L. flavum*	51800	144102^a^	http://mus.utu.fi/TFU.207260 ^P^	KFL1315NDB	*Q. robur*	Tree wound	Wierzchosławice, PL	MH055543	MH055572	MH055600	MH055629	MH496063	MH496090
*L. flavum*				KFL114916NDB	*Q. robur*	Tree wound	Ispina, PL	MH055542	MH055571	MH055599	MH055628	MH496062	MH496089
*L. flavum*	51801			KFL114416NDB	*Q. robur*	Tree wound	Ispina, PL	MH055541	MH055570	MH055598	MH055627	MH496061	MH496088
*L. flavum*				KFL24516NDB	*Q. robur*	Tree wound	Babimost, PL	MH055545	MH055574	MH055602	MH055631	MH496065	MH496092
*L. flavum*	51802	144103		KFL14516NDBCZ	*Q. rubra*	Tree wound	Wierzchosławice, PL	MH055544	MH055573	MH055601	MH055630	MH496064	MH496091
*L. flavum*				KFL111916NDB	*Q. robur*	Tree wound	Wierzchosławice, PL	MH055540	MH055569	MH055597	MH055626	MH496060	MH496087
*L. flavum*	51803	144104		KFL111316NDB	*Q. robur*	Tree wound	Wierzchosławice, PL	MH055539	MH055568	MH055596	MH055625	MH496059	MH496086
*L. flavum*	51804	144105	http://mus.utu.fi/TFU.207261 ^P^	KFL103416XD	*Q. robur*	*X. dispar*	Resko, PL	MH055537	MH055566	MH055594	MH055623	MH496057	MH496084
*L. flavum*	51805	144106^a^		KFL104316XD	*Q. robur*	*X. dispar*	Resko, PL	MH055538	MH055567	MH055595	MH055624	MH496058	MH496085
*O. brevicolle*		150.78		WIN(M)811	*P. tremuloides*	Beetle gallery		MH055549	AF155670	MH055606	MH055635	MH496069	MH496096

^1^CMW Culture Collection of the Forestry and Agricultural Biotechnology Institute (FABI), University of Pretoria, Pretoria, South Africa; CBS Westerdijk Fungal Biodiversity Institute, Utrecht, The Netherlands; TFU the TUR Fungus collections of the Turku University, Finland; KFL Culture collection of the Department of Forest Pathology, Mycology and Tree Physiology; University of Agriculture in Krakow, Poland; N Culture Collection at Norwegian Institute of Bioeconomy, Norway, WIN the University of Manitoba (Winnipeg) Collection Colorado, Larimer County, Roosevelt Nat. Forest, Redfeather District, USA

^2^Host species: *A. incana*—*Alnus incana*, *F. sylvatica*—*Fagus sylvatica*, *Q. robur*—*Quercus robur*, *Q. rubra*—*Quercus rubra*

^3^Beetle species: *T. domesticum*—*Trypodendron domesticum*, *D. alni*—*Dryocoetes alni*, *T. signatum*—*Trypodendron signatum*, *A. dispar*—*Anisandrus dispar*

^a^Isolates used in growth and morphological studies; ^P^ex-paratype; ^H^ex-holotype

### DNA extraction, PCR and sequencing

Fungal isolates were grown on 2% malt extract agar [MEA: 20 g Bacto™Malt Extract^−1^, 20 g BBL™Agar, Grade A^−1^ (Becton, Dickinson and Company Sparks, USA) and 1 L distilled water] in 60 mm plastic Petri dishes for 1–2 weeks prior to DNA extraction. DNA was extracted using the Genomic Mini AX Plant Kit (A&A Biotechnology, Gdynia, Poland) according to the manufacturer’s protocol.

Six loci were amplified for sequencing and phylogenetic analyses, including ITS1–5.8 S–ITS2, ITS2–LSU, ACT, βT, CAL and TEF 1-α. The following primers were used: ITS 1-F (Gardes and Bruns 1993) and ITS4 (White et al. 1990) for ITS1–5.8 S–ITS2, ITS3 and LR3 (White et al. 1990) for ITS2-LSU, Lepact-F and Lepact-R (Lim et al. 2004) for ACT, Bt2a and Bt2b (Glass and Donaldson 1995) plus T10 (O’Donnell and Cigelnik 1997) for βT, CL2F and CL2R (Duong et al. 2012) for CAL, and EF1-F and EF2-R (Jacobs et al. 2004) for TEF 1-α.

Amplification of the gene regions was performed under the following conditions: a denaturation step at 98 °C for 30 s followed by 35 cycles of 5 s at 98 °C, 10 s at 52–64 °C (depending on the type of primer and fungal species) and 30 s at 72 °C, and a final elongation step at 72 °C for 8 min. Gene fragments were amplified in a 25 µL reaction mixture containing 0.25 µL of Phusion High-Fidelity DNA polymerase (Finnzymes, Espoo, Finland), 5 µL Phusion HF buffer (5×), 0.5 µL of dNTPs (10 mM), 0.75 µL DMSO (100%) and 0.5 µL of each primer (25 µM). Amplification reactions were performed in the LabCycler Gradient (Sensoquest Biomedical Electronics GmbH, Germany). The PCR products were visualized under UV light on a 2% agarose gel stained with Midori Green DNA Stain (Nippon Genetic Europe).

Amplified products were sequenced with the BigDye^®^ Terminator v 3.1 Cycle Sequencing Kit (AB Applied Biosystems, Foster City, CA, USA) and ABI PRISM 3100 Genetic Analyzer (Applied Biosystems, Foster City, USA), at the DNA Research Centre (Poznań, Poland) using the same primers that were used for the PCR. The sequences (Table 1) were deposited in NCBI GenBank and compared with sequences in GenBank using the BLASTn algorithm.

### Sequence analyses

**Fig. 3:**
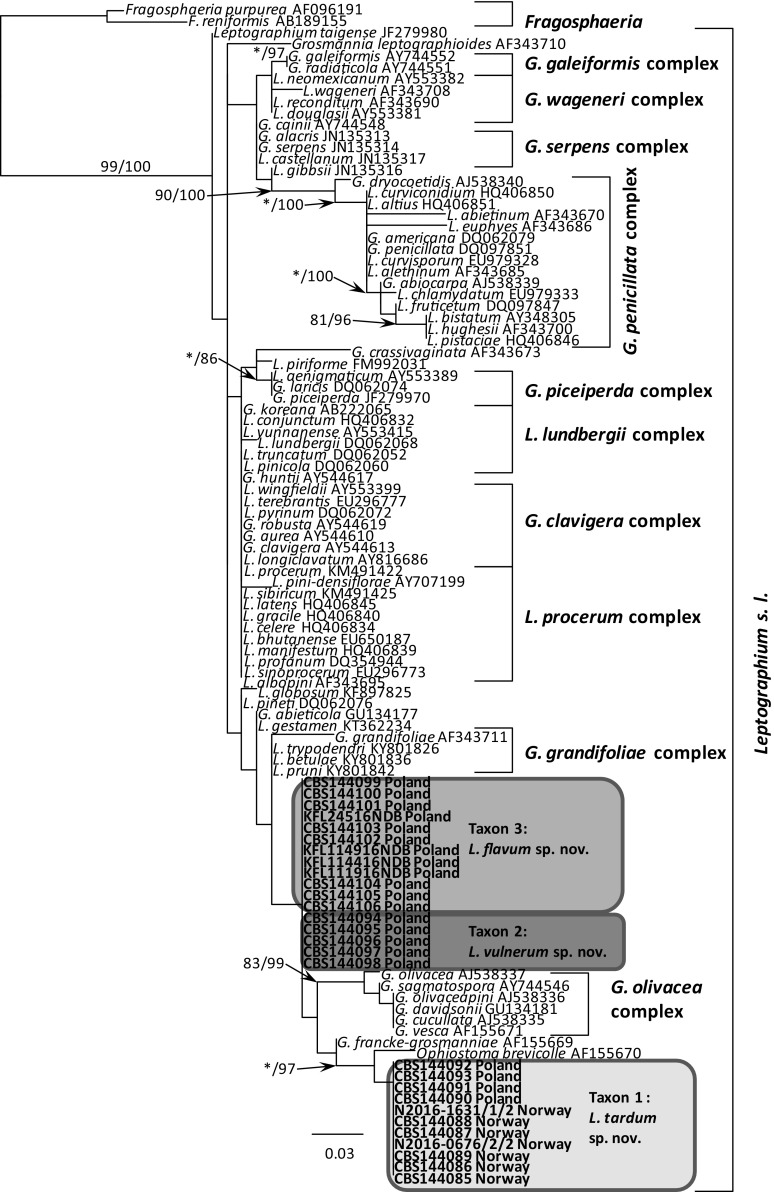
Phylogram obtained from Maximum Likelihood (ML) analyses of the LSU region showing the placement of isolates obtained from Poland and Norway in *Leptographium s. l.* Sequences obtained during this study are presented in bold type. Bootstrap values > 75% for ML and posterior probabilities > 75% obtained from Bayesian (BI) analyses are indicated at the nodes as follows: ML/BI. *Bootstrap values < 75%. The tree is drawn to scale with branch length measured in the number of substitutions per site

**Fig. 4:**
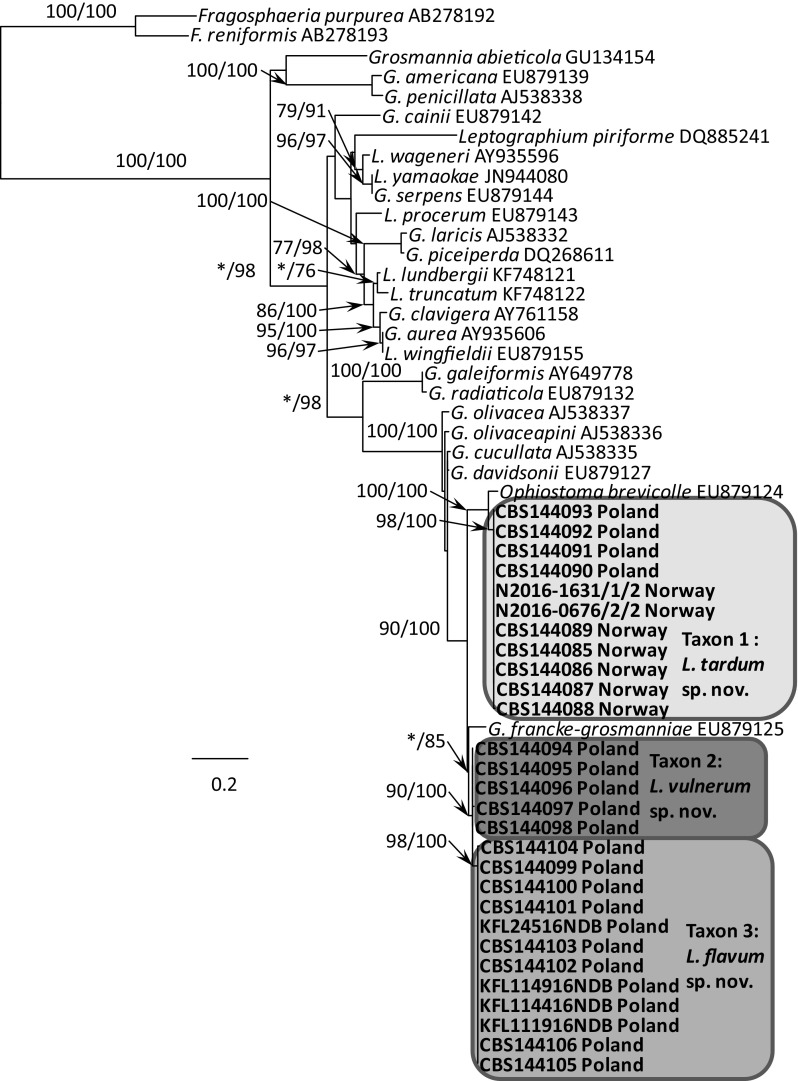
Phylogram obtained from ML analyses of the ITS1–5.8 S–ITS2 region showing the placement of isolates obtained from Poland and Norway in *Leptographium s. l.* Sequences obtained during this study are presented in bold type. Bootstrap values > 75% for ML and posterior probabilities > 75% obtained from Bayesian (BI) analyses are presented at the nodes as follows: ML/BI. *Bootstrap values < 75%. The tree is drawn to scale with branch length measured in the number of substitutions per site

**Fig. 5:**
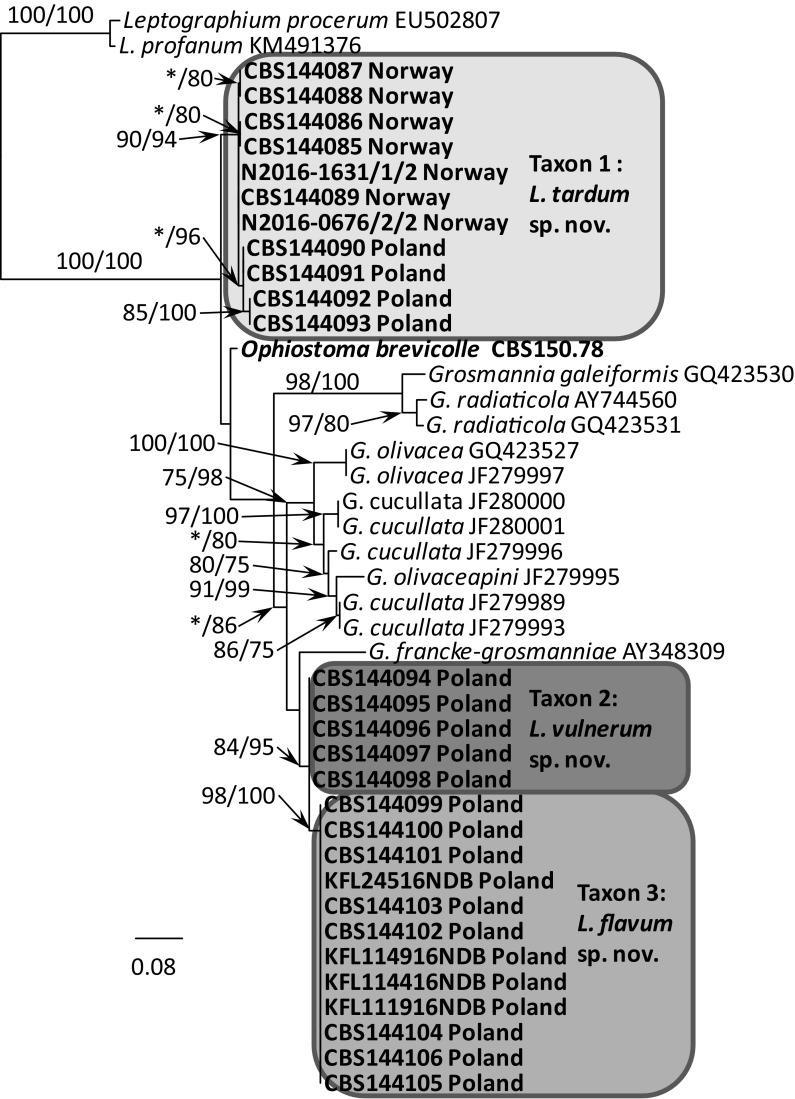
Phylogram obtained from the analysis of βT sequences for members of *Leptographium s. l.* showing the phylogenetic relationships of the Polish and Norwegian isolates collected during this study. Sequences obtained during this study are presented in bold type. The phylogram was obtained from Maximum Likelihood (ML) analyses. Bootstrap values > 75% for ML and posterior probabilities > 75% obtained from Bayesian (BI) analyses are presented at the nodes as follows: ML/BI. *Bootstrap values < 75%. The tree is drawn to scale with branch length measured in the number of substitutions per site

**Fig. 6:**
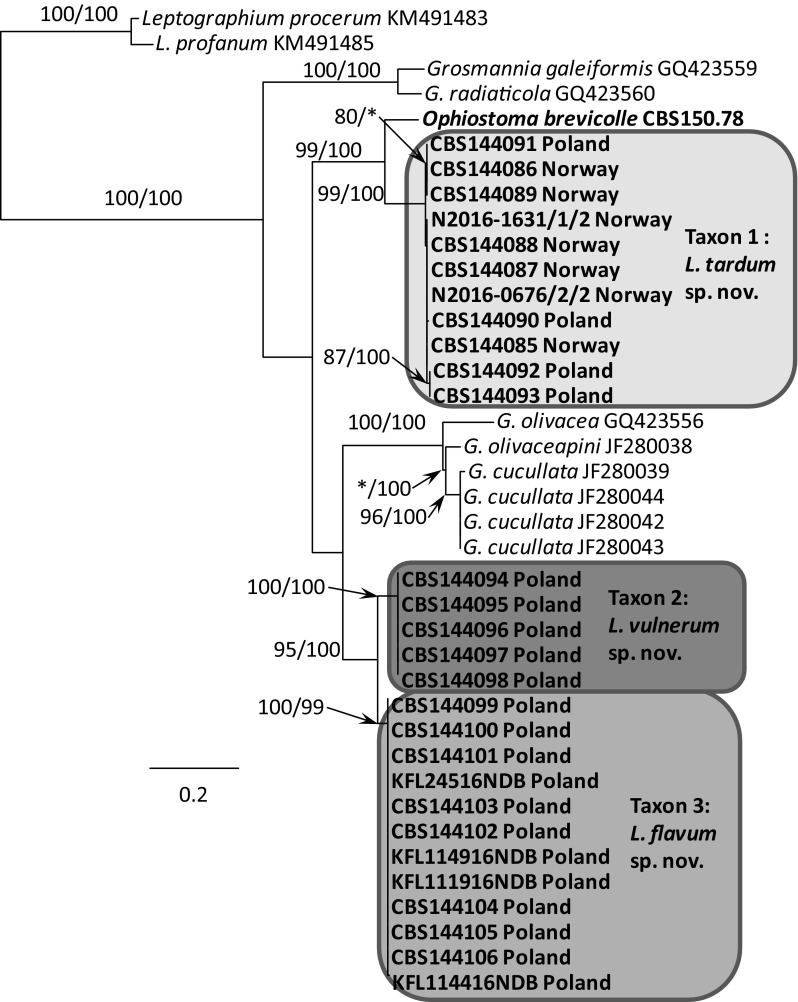
Phylogram obtained from the analysis of TEF1-α sequences for members of *Leptographium s. l.* showing possible placements for the Polish and Norwegian isolates examined in this study. Sequences obtained during this study are presented in bold type. The phylogram was obtained from Maximum Likelihood (ML) analyses. Bootstrap values > 75% for ML and posterior probabilities > 75% obtained from Bayesian (BI) analyses are presented at nodes as follows: ML/BI. *Bootstrap values < 75%. The tree is drawn to scale with branch length measured in the number of substitutions per site

**Fig. 7:**
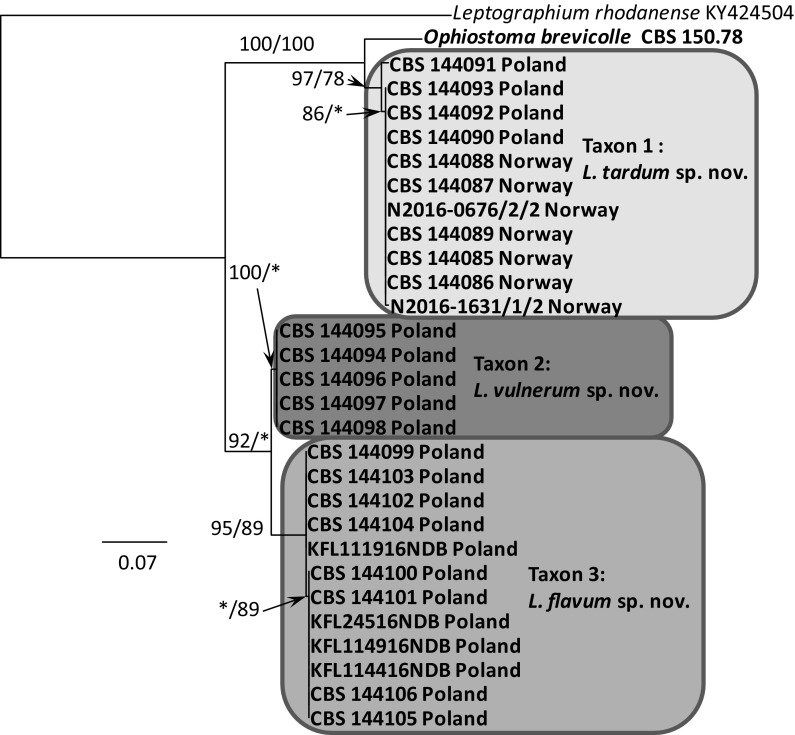
Phylogram obtained from the analysis of ACT sequences for members of *Leptographium s. l.* showing possible placements for the Polish and Norwegian isolates examined in this study. Sequences obtained during this study are presented in bold type. The phylogram was obtained from Maximum Likelihood (ML) analyses. Bootstrap values > 75% for ML and posterior probabilities > 75% obtained from Bayesian (BI) analyses are presented at nodes as follows: ML/BI. *Bootstrap values < 75%. The tree is drawn to scale with branch length measured in the number of substitutions per site

**Fig. 8:**
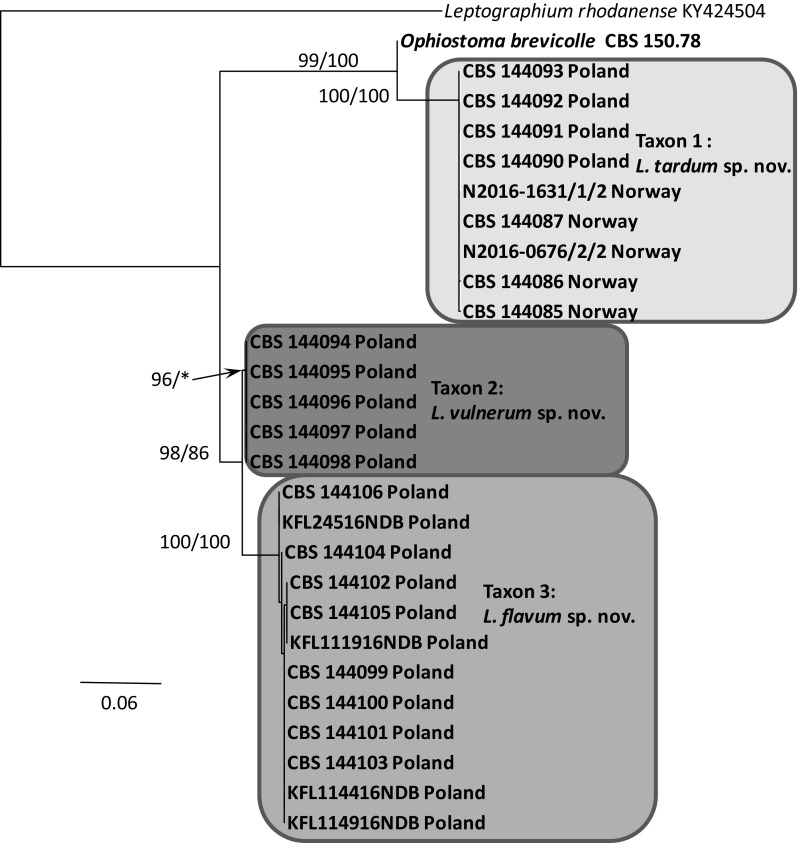
Phylogram obtained from the analysis of CAL sequences for members of *Leptographium s. l.* showing possible placements for the Polish and Norwegian isolates examined in this study. Sequences obtained during this study are presented in bold type. The phylogram was obtained from Maximum Likelihood (ML) analyses. Bootstrap values > 75% for ML and posterior probabilities > 75% obtained from Bayesian (BI) analyses are presented at nodes as follows: ML/BI. *Bootstrap values < 75%. The tree is drawn to scale with branch length measured in the number of substitutions per site

BLAST searches using the BLASTn algorithm were performed to retrieve similar sequences from GenBank (http://www.ncbi.nlm.nih.gov). Accession numbers of these sequences are presented in the corresponding phylogenetic trees (Figs. 3, 4, 5, 6, 7, 8). Newly obtained sequences were deposited in GenBank and their accession numbers are presented in Table 1.

Individual data sets for the ITS1–5.8 S–ITS2, ITS2-LSU, ACT, βT, CAL, and TEF1-α gene regions were used for phylogenetic analyses. Data sets were compiled and edited with programs implemented within the Molecular Evolutionary Genetic Analysis (MEGA) v6.06 software (Tamura et al. 2013). The ITS2-LSU sequences obtained from isolates of our newly described *Leptographium* species (Table 1) were compared with those of 75 other *Leptographium s. l.* species. The later sequences were obtained from GenBank and allowed for inferring the phylogenetic position of these new species within the genus. In addition the ITS1–5.8 S–ITS2 and four protein coding gene regions (ACT, βT, CAL, TEF1-α) for 28 isolates (Table 1) were sequenced to enhance the delineation of closely related species.

Sequence alignments were performed using the online version of MAFFT v7 (Katoh and Standley 2013). The ITS, LSU, ACT, βT, CAL and TEF-1α data sets were aligned using the E-INS-i strategy with a 200PAM/κ = 2 scoring matrix, a gap opening penalty of 1.53 and an offset value of 0.00. Aligned data sets of the protein-coding genes were compared to gene maps constructed by Yin et al. (2015) to determine the presence or absence of introns and confirm that introns and exons were appropriately aligned (Tables S1–S4).

Phylogenetic analyses were performed for each of the data sets using two different methods: maximum likelihood (ML) and Bayesian inference (BI). For ML and Bayesian analyses, the best-fit substitution models for each data set were established using the corrected Akaike Information Criterion (AICc) in jModelTest 2.1.10 (Guindon and Gascuel 2003; Darriba et al. 2012). Maximum likelihood (ML) analyses were conducted with PhyML 3.0 (Guindon et al. 2010), via the Montpelier online server (http://www.atgc-montpellier.fr/phyml/) with 1000 bootstrap replicates. BI analyses based on a Markov Chain Monte Carlo (MCMC) were carried out with MrBayes v3.1.2 (Ronquist and Huelsenbeck 2003). The MCMC chains were run for 10 million generations using the best-fit model. Trees were sampled every 100 generations, resulting in 100,000 trees from both runs. The burn-in value for each dataset was determined in Tracer v1.4.1 (Rambaut and Drummond 2007). The remaining trees were utilized to generate a majority rule consensus tree for determining the posterior probability values.

### Morphological characterization

Morphological observations were made for selected isolates and herbarium specimens chosen to represent the type specimens. Cultures were grown on 2% MEA with or without host tree twigs to induce potential ascocarp formation. The autoclaved twigs with bark were placed in the middle of the agar plates. Fungal cultures were grown starting with a single spore, and crossings were made for all isolates following the technique described by Grobbelaar et al. (2010). Cultures were incubated at 25 °C for 14–21 days and inspected frequently for the formation of fruiting structures.

Morphological characteristics were examined by mounting the sexual and asexual fruiting structures in 80% lactic acid on glass slides, and these were observed using a Nikon Eclipse 50*i* microscope (Nikon^®^ Corporation, Tokyo, Japan) with an Invenio 5S digital camera (DeltaPix^®^, Maalov, Denmark) to capture photographic images. Microscopy was done as described by Kamgan Nkuekam et al. (2011a, b). Colours were described with the charts of Kornerup and Wanscher (1978).

Fifty measurements were made for each significant taxonomically relevant structure whenever possible, with the Coolview 1.6.0 software (Precoptic^®^, Warsaw, Poland). Averages, ranges and standard deviations were computed for the measurements, and these are presented in the format ‘(min–max) (mean–SD)’.

### Culture characteristics

Growth characteristics for the three newly described species (Taxon 1 to 3) were determined by analysing the radial growth for four representative isolates of each of the studied species (Table 1). Agar disks 5 mm diam. were cut from actively growing margins of colonies of each isolate to be tested, and placed at the center of plates containing 2% MEA. Four plates for each isolate were incubated at the following temperatures: 5, 10, 15, 20, 25, 30 and 35 °C. Colony diameters (three measurements per plate) were determined 7 and 14 d after inoculation and growth rates were calculated as mm/d.

## Results

### Morphological characteristics

Isolates of the three new taxa emerging from this study were dissimilar in growth and culture morphology. Colonies for Taxon 1 displayed a rusty-yellowish colour; whereas Taxon 2 colonies had a greyish appearance and those of Taxon 3 were deep yellowish. The growth of the three new taxa on MEA was also different, with Taxon 1 being the slowest and Taxon 2 being the fastest. The optimal growth temperature was 25 °C for isolates of Taxon 1 and 20 °C for isolates of Taxon 2 and Taxon 3. For all new taxa examined the mononematous conidiophores with dark olivaceous stipes were common and hyphae were superficial on the agar. Taxon 2 and Taxon 3 produced shorter mononematous conidiophores with light olivaceous stipes. The dimensions of most morphological structures were similar and partly overlapped among species in this complex. The droplets containing conidia, appeared initially hyaline, but turned whitish-yellowish with age. A sexual state could be induced in all isolates, the most distinct feature observed in both the herbarium specimens and the studied isolates were the pale brown, straight and sharply pointed ostiolar hyphae and orange-section shaped ascospores with cucullate gelatinous sheaths. Isolates derived from single spores for all three new taxa produced ascomata in culture, suggesting that they are homothallic. Morphological differences that distinguish the three newly described species are discussed in the *Notes* within the Taxonomy section.

### DNA sequence analyses

The amplified DNA fragments were 563–655 bp long for the ITS1–5.8 S–ITS2 region, 936–971 bp long for the 5.8S-ITS2-LSU region, 308–437 bp long for the partial βT, 812–861 bp long for the TEF1-α segment, 835–884 bp long for the partial ACT and 500–544 bp long for the partial CAL. The aligned data set for the ITS1–5.8S–ITS2 region included 105 taxa and 652 characters (with gaps). The aligned data set for the LSU gene region included 105 taxa and 334 characters (with gaps). The βT data set consisted of 43 taxa and 380 characters (with gaps), and included the partial sequences for exon 3/4, intron 4 and partial sequences for exon 5/6. The TEF-1α data set consisted of 39 taxa and 811 characters (with gaps), including partial sequences for exon 4, all of intron 4, exon 5, intron 5, and partial sequences for exon 6. The ACT data set consisted of 30 taxa and 816 characters (with gaps), and included the partial sequences for exon 5, intron 5 and partial sequences for exon 6. The CAL data set consisted of 28 taxa and 634 characters (with gaps), including partial sequences for exon 3, all of intron 3, exon 4, intron 4, exon 5/6, and partial sequences for intron 6. The BI and ML analyses for each data set produced trees with similar topologies (Figs. 3–8). The best-fitting substitution models selected for ML/BI analyses were GTR + I+G, HKY + I, HKY + G, HKY + G, GTR + I+G and GTR + I for respectively the ITS, LSU, βT, TEF-1α, ACT and CAL data sets.

The phylogenetic trees arising from the analyses of the LSU data for members of *Leptographium s. l.* showed sequences representing the *G. olivacea* complex being positioned between sequences that represent Taxon 1 and Taxa 2–3 with some nodes receiving statistical support (Fig. 3). In the LSU tree, 11 isolates of Taxon 1 originating from Norway and Poland formed a clade that included the sequence of the ex-type isolate of *O. brevicolle*, while 17 isolates of Taxon 2 and Taxon 3 resided in a separate clade. However, the LSU data did not distinguish clearly between members of Taxon 2 and Taxon 3. The LSU sequence for *G. francke*-*grosmanniae* branched basal to *O. brevicolle* and therefore outside of the *G. olivacea* species complex (Fig. 3).

ITS sequences obtained for our isolates when compared with sequences obtained from GenBank and reference isolates (Fig. 4) confirmed that our isolates could be assigned into three distinct taxa that are positioned adjacent to the *G. olivacea* species complex. Phylogenetic analysis of the ITS sequences showed that Taxon 1 is closely related to *O. brevicolle* while Taxon 2 and Taxon 3 appear to be more closely affiliated with *G. francke*-*grosmanniae*. Unlike the LSU sequences, the ITS data provides some differentiation between isolates of Taxon 2 and Taxon 3 (Fig. 4).

In the ß-tubulin tree (Fig. 5), isolates of Taxon 1 grouped within a well-supported distinct lineage adjacent to the species that represent the *G. olivacea* complex and *O. brevicolle*. Intraspecific sequence variation of the ßT gene was found within this taxon, especially between the Norwegian and Polish isolates. Intraspecific variability detected for members of Taxon 1 ranged up to a maximum of 5 nucleotide positions (Table S1). A second group of isolates representing Taxon 2 and Taxon 3 grouped with *G. francke*-*grosmanniae* in the ß-tubulin based phylogenetic tree (Fig. 5), but differed in length by 38 and 41 bp nucleotide positions from that species. Analyses of the partial βT gene also distinguished clearly between members comprising Taxon 2 versus members of Taxon 3 (Fig. 5).

The phylogram based on the TEF-1α data confirmed that Taxon 1 and Taxa 2–3 form two well-supported monophyletic lineages adjacent to the species that represent the *G. olivacea* complex (Fig. 6). Minor intraspecific sequence variation was found, but only among isolates of Taxon 1, up to a maximum of 5 positions (Table S2).

The phylogram obtained from for the ACT gene region showed differences between Taxa 1–3 and *O. brevicolle* (Fig. 7). Minor intraspecific sequence variation was found among isolates of Taxon 1 (up to a maximum of 4 positions, Table S3) and Taxon 3 (up to a maximum of 1 position, Table S3).

In the CAL tree (Fig. 8), isolates of Taxa 1–3 and *O. brevicolle* grouped into four well-supported distinct lineages. Intraspecific variability was detected only for members of Taxon 3 which ranged up to a maximum of 10 nucleotide positions (Table S4).

## Taxonomy

Based on DNA sequences and morphological differences, Taxon 1 with isolates from Poland and Norway, and Taxa 2 and 3 from Poland could be distinguished from other *Leptographium* species, and are thus described here as new species.

### Taxon 1

**Fig. 9:**
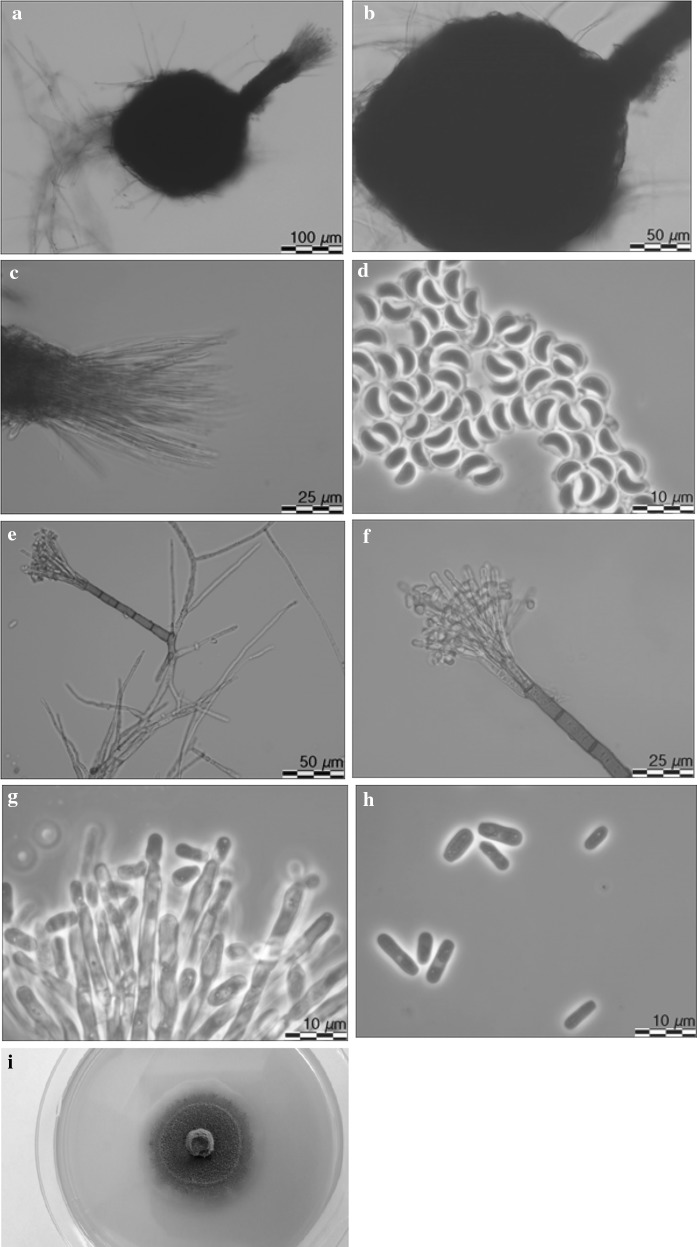
Morphological characteristics of *Leptographium tardum* sp. nov. (CBS 144091). **a** ascoma, **b** ascomatal base, **c** ostiolar hyphae, **d** ascospores, **e** and **f** conidiophore, **g** conidiogenous apparatus, **h** conidia, **i** fourteen day old culture on MEA

*Leptographium tardum* T. Aas, H. Solheim and R. Jankowiak, sp. nov. (Fig. 9) MycoBank: 826759.

*Etymology* Name refers to the very slow growth of this fungus on malt agar.

*Sexual state* develops on malt agar in 21 days. *Perithecia* superficial on wood and agar, bases light brown to dark brown, globose, unornamented, 100–238 (mean 163.7 ± 23.8) μm in diameter, necks dark brown, cylindrical, straight, 122–314.8 (mean 196.1 ± 31.6) μm long (including ostiolar hyphae), 23.3–46.9 (mean 33.9 ± 5.5) μm wide at base, 25.8–42.4 (mean 34 ± 4.7) μm wide at the tip (Fig. 9a, b). *Ostiolar hyphae* present, pale brown, straight, septate, numerous, divergent, tapering at the tip, up to 151.6 μm long (Fig. 9c). *Asci* not seen. *Ascospores* (Fig. 9d) one-celled, hyaline, orange section shaped in side view, ellipsoidal in face view, globose in end view, 4.3–5.9 (mean 5.1 ± 0.41) × 2.3–4.6 (mean 3.2 ± 0.49) μm including hyaline gelatinous sheath, 0.1–1 μm thick.

*Conidiophores* macronematous, arising directly from hyphae, single solitary, without rhizoidal hyphae at the bases but often from the base emerged new conidiophores in different directions, 107.5–209.2 (mean 161.5 ± 19.8) μm in length (Figs. 9e, f). *Stipes* erect, olivaceous, 3–9 septate, 76.3–150.5 (mean 106.5 ± 19.2) μm long (from first basal septum to below primary branches), 4.4–7.8 (mean 5.6 ± 0.7) μm wide below primary branches, apical cell not swollen, 5.4–8.2 (mean 6.7 ± 0.7) μm wide at base, basal cell not swollen. Asexual state: *Conidiogenous apparatus* 39.6–68.9 (mean 51.1 ± 7.6) μm long (excluding conidial mass) consisting of 2–4 series of branches-type B (more than two branches) (Jacobs and Wingfield 2001) (Fig. 9g). Primary branches dark olivaceous, cylindrical, smooth, 11.5–19.5 × 2.8–5.5 μm. *Conidiogenous cells* hyaline, tapering from base to apex, 14.1–25.7 (mean 18.4 ± 2.4) × 1.9–2.6 (mean 2.1 ± 0.15) μm. *Conidia* (Fig. 9h) hyaline, ellipsoidal to cylindrical, 3.9–8.1 (mean 5.6 ± 0.91) × 1.2–2.9 (mean 1.9 ± 0.36) μm, accumulating around the conidiogenous apparatus as a light yellow mucilaginous mass.

*Cultural characteristics* Colonies on MEA hyaline at first, becoming light rusty and later darker, mycelium appressed and immersed (Fig. 9i). Colony margin effuse. Hyphae amber yellow in colour (Kornerup and Wanscher 1978), smooth, slightly constricted at the septa, 1.7–6.1 (mean 3.4 ± 0.9) µm diam. Perithecia and *Leptographium* asexual morph co-occur in culture. Optimal growth temperature is 25 °C, radial growth rate 1.5 (± 0.1) mm/d, growth reduced at 10 and 15 °C, no growth at 5 and 35 °C.

Host trees: *Alnus incana*, *Fagus sylvatica*, *Fraxinus exselsior*, *Quercus robur*
Insect vector: *Trypodendron domesticum*, *T. signatum*, *Dryocoetes alni*
Distribution: Norway, Poland

*Type material* POLAND, Rozpucie, from *Trypodendron domesticum* beetle infesting *Fagus sylvatica*, 29 August 2015, *R. Jankowiak*, holotype TUR http://mus.utu.fi/TFU.207251, culture ex-holotype CBS 144091 = CMW 51789; POLAND, Zabierzów, from *Trypodendron domesticum* beetle infesting *Fagus sylvatica*, 24 January 2014, *R. Jankowiak*, paratype TUR http://mus.utu.fi/TFU.207252, culture ex-paratype CBS 144092 = CMW 51790; NORWAY, Troms, from *Trypodendron signatum* beetle infesting *Alnus incana*, 27 September 2016, *G. Kvammen*, paratype TUR http://mus.utu.fi/TFU.207249, culture ex-paratype CBS 144087 = CMW 51784.

*Notes* Isolates of *L. tardum* grouped close to *O. brevicolle* in the phylogenetic analyses of the LSU and ITS sequences (Figs. 3, 4), however they can clearly be separated from this fungus based on sequences of the four protein-coding genes (Figs. 5, 6, 7, 8).

Morphologically, *L. tardum* differs from *O. brevicolle* in having larger ascospores and conidia, and the presence of ostiolar hypha on the ascomatal neck. In addition, *L. tardum* produces rusty-yellowish cultures in contrast to the olivaceous colored colonies of *O. brevicolle*. The optimal growth on MEA for *L. tardum* is 25 °C while for *O. brevicolle* optimal growth is at 30 °C. Morphologically, *L. tardum* is more similar to *G. francke*-*grosmanniae.* However, *L. tardum* produces larger ascospores and conidia, condiophores are without rhizoids, and colonies are not olivaceous.

*Leptographium tardum* was isolated from various beetle species on *A. incana* in two sample plots in south-eastern Norway and one area in northern Norway. At these sites among the following beetles sampled *L. tardum* was recovered from *T. signatum*, *T. domesticum*, and *D. alni* 95, 7, and 3% respectively. *Leptographium tardum* was also recorded on *T. domesticum* recovered from *F. excelsior* and *Q*. *robur* on the same sample plots. In contrast to Norway, *L. tardum* was rarely found in association with *T. domesticum* from *F. sylvatica* in Poland. It was isolated from 1% of the beetles collected from *F. sylvatica* logs.

### Taxon 2

**Fig. 10:**
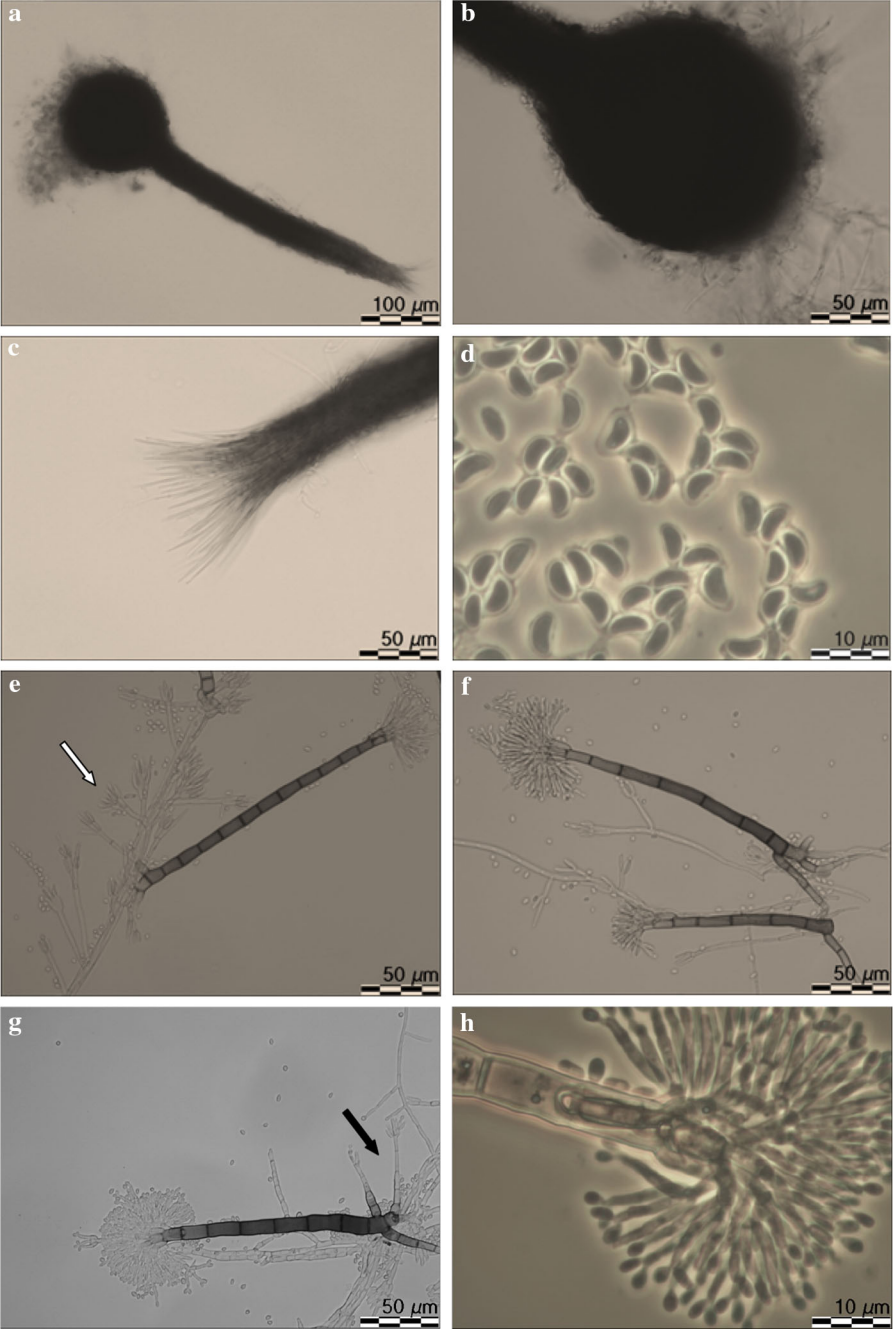

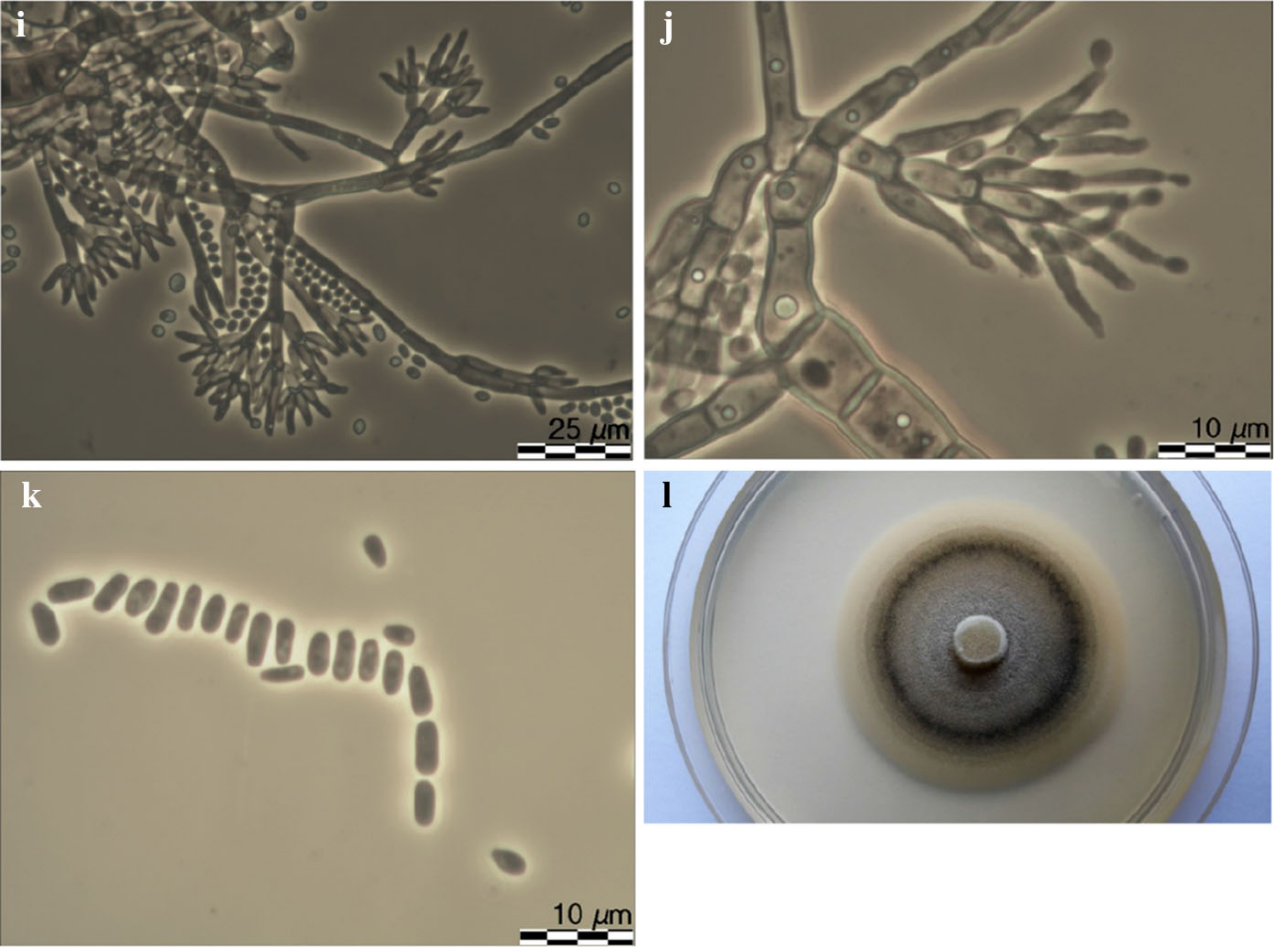
Morphological characteristics of *Leptographium vulnerum* sp. nov. (CBS 144096). **a** ascoma, **b** ascomatal base, **c** ostiolar hyphae, **d** ascospores, **e**–**g** conidiophores (type 1), white arrow indicates conidiophores of type 2, black arrow indicates secondary conidiophores emerging from base of primary conidiophores, **h** conidiogenous apparatus of conidiophores type 1, **i** conidiophores (type 2), **j** conidiogenous apparatus of conidiophore type 2, **k** conidia, **l** fourteen day old culture on MEA

*Leptographium vulnerum* R. Jankowiak and A. Ostafińska, sp. nov. (Fig. 10). MycoBank: 826760.

*Etymology* Name refers to wound of tree where the holotype and other specimens were collected.

*Sexual state* develops on malt agar in 21 days. *Perithecia* superficial on wood and agar, bases black, globose, unornamented, 123.4–226,1 (mean 177.4 ± 28.6) μm in diameter, necks dark brown, cylindrical, straight, 341–617.7 (mean 455.3 ± 85.1) μm long (including ostiolar hyphae), 39–77 (mean 48.7 ± 9.5) μm wide at base, 24.7–42.6 (mean 35.3 ± 4.8) μm wide at the tip (Fig. 10a, b). *Ostiolar hyphae* present, pale brown, straight, septate, numerous, divergent, tapering at the tip, up to 116 μm long (Fig. 10c). *Asci* not seen. *Ascospores* one-celled, hyaline, orange section shaped in side view, ellipsoidal in the face view, globose in end view, 4.2–6.1 (mean 5.1 ± 0.38) × 1.9–3.5 (mean 2.5 ± 0.4) μm including hyaline gelatinous sheath, 0.2–0.7 μm thick (Fig. 10d).

*Asexual states*: (1): *Conidiophores* (type 1): macronematous, arising directly from hyphae, without rhizoidal hyphae at the bases but often secondary conidiophores emerge from the base of primary conidiophores, 119–170 (mean 142.4 ± 12.3) μm in length (Figs. 10e–g). *Stipes* erect, olivaceous, 3–14 septate, 79–117 (mean 95.2 ± 12.3) μm long from first basal septum to below primary branches, 5.3–8.7 (mean 6.6 ± 0.7) μm wide below primary branches, apical cell not swollen, 6.3–9.5 (mean 7.9 ± 0.7) μm wide at base, basal cell not swollen. *Conidiogenous apparatus* 39.4–71.1 (mean 48.8 ± 6.6) μm long (excluding conidial mass) consisting of (1–) 2 (–3) series of branches-type B (more than two branches) (Jacobs and Wingfield 2001) (Fig. 10h). Primary branches dark olivaceous, cylindrical, smooth, 10.3–17.2 × 2.6–5.7 μm. *Conidiogenous cells* hyaline, tapering from base to apex, 11.9–24 (mean 16.6 ± 2.4) × 1.6–2.7 (mean 2.2 ± 0.3) μm. *Conidia* hyaline, oblong to elliptical, sometimes obovate (Fig. 10h), 2.7–5.1 (mean 3.9 ± 0.47) × 0.8–1.9 (mean 1.5 ± 0.2) μm, accumulating around the conidiogenous apparatus in a creamy mucilaginous mass.

(2): *Conidiophores* (type 2): occurring singly or in groups, mostly on aerial mycelia or emerge from the base of conidiophores as described above, macronematous, mononematous, 24.4–41.8 (mean 31.6 ± 4.7) μm in length including the conidial mass, rhizoid like structures absent (Figs. 8e, i). Stipes hyaline or light olivaceous, simple, 1–4 septate, 13.2–26.7 (mean 18.3 ± 3.0) μm long, 1.3–2.9 (mean 2 ± 0.3) μm wide below primary branches. Conidiogenous apparatus with 1–2 series of cylindrical branches (Fig. 10j). Primary branches, 2–4, light olivaceous, smooth, cylindrical aseptate, 5.7–20.3 (mean 11.3 ± 3.2) μm long, 1.2–2.8 (mean 2 ± 0.4) μm wide arrangement of the primary branches on the stipe-type B (more than two branches) (Jacobs and Wingfield 2001). Conidiogenous cells discrete, 2–4 per branch, cylindrical, tapering slightly at the apex, 4.5–12.8 (mean 7.9 ± 1.7) μm long and 0.7–2.6 (mean 1.5 ± 0.4) μm wide. *Conidia* hyaline, oblong to elliptical, sometimes obovate, 1.4–2.8 (mean 2 ± 0.35) × 0.8–2.2 (mean 1.5 ± 0.26) μm.

*Cultural characteristics*: Colonies on MEA hyaline at first, later becoming light greyish in the centre, with a darker edge; mycelium appressed and immersed (Fig. 10l). Colony margin smooth. Hyphae hyaline or pale grey in colour (Kornerup and Wanscher 1978), smooth, submerged in the medium and aerial mycelium sparse, not constricted at the septa, 0.9–3.4 (mean 1.9 ± 0.6) µm diam.

Perithecia and asexual morphs co-occur in culture. Optimal growth temperature is 20 °C, radial growth rate 2.4 (± 0.1) mm/d, growth reduced at 25 °C, no growth at 5, 30 and 35 °C.

Host trees: *Carpinus betulus*, *Fagus sylvatica*, *Quercus robur*, *Quercus rubra*
Insect vector: unknown
Substrate: natural fresh lesions (necrosis) on tree stem
Distribution: Poland

*Type material* POLAND, Babimost, from *Fagus sylvatica* wood, 26 May 2016, *R*. *Jankowiak*, holotype TUR http://mus.utu.fi/TFU.207255, culture ex-holotype CBS 144096 = CMW 51794; POLAND, Babimost, from *Carpinus betulus* wood, 26 May 2016, *R*. *Jankowiak*, paratype TUR http://mus.utu.fi/TFU.207254, culture ex-paratype CBS 144094 = CMW 51792; POLAND, Wierzchosławice, from *Quercus rubra* wood, 13 October 2016, *R. Jankowiak*, paratype TUR http://mus.utu.fi/TFU.207256, culture ex-paratype CBS 144097 = CMW 51795.

*Notes* Isolates of *L. vulnerum* grouped close to *L. flavum* in the phylogenetic analyses of the ITS and LSU sequences (Figs. 3, 4), but can be clearly separated based on sequences of the four protein-coding genes (Figs. 5–8). These two species differ in 4 bp nucleotide positions with regards to β-tubulin (Table S1) and by 31 bp with regards to the TEF-1α sequence (Table S2). The phylogenetic analysis of the ITS and ßT sequences showed that *L. vulnerum* and *L. flavum* are closely related to *G. francke*-*grosmanniae* (Figs. 4, 5).

Morphologically, *L. vulnerum* differs from *G. francke*-*grosmanniae* in having larger perithecial bases and necks, larger ascospores and the presence of conidiophores without rhizoids. *Leptographium vulnerum* produces two types of conidiophores: large darkly pigmented conidiophores and non-pigmented conidiophores with short stipes. In addition, *L. vulnerum* generates greyish cultures and the optimal growth on MEA is at 20 °C.

Morphologically, *L. vulnerum* is most similar to *L. flavum.* Both species produce two types of conidiophores. The greatest differences between these species with regarding to morphology are that *L. vulnerum* produces larger perithecial bases and necks, and larger ascospores. Furthermore, *L. vulnerum* grows slowly at temperatures that exceed 20 °C, while growth of *L. flavum* is only slightly reduced at 25 °C. In contrast to *L. flavum*, no growth of *L. vulnerum* is observed at 30 °C. *Leptographium vulnerum* produces greyish cultures, while *L. falvum* forms deep yellowish cultures with clear rings.

*Leptographium vulnerum* was isolated from lesions on the following hardwoods respectively in 8, 6, 4, and 0.7% in six sample plots in southern Poland: *Q. rubra*, *C. betulus*, *Q. robur*, and *F. sylvatica.*

### Taxon 3

**Fig. 11:**
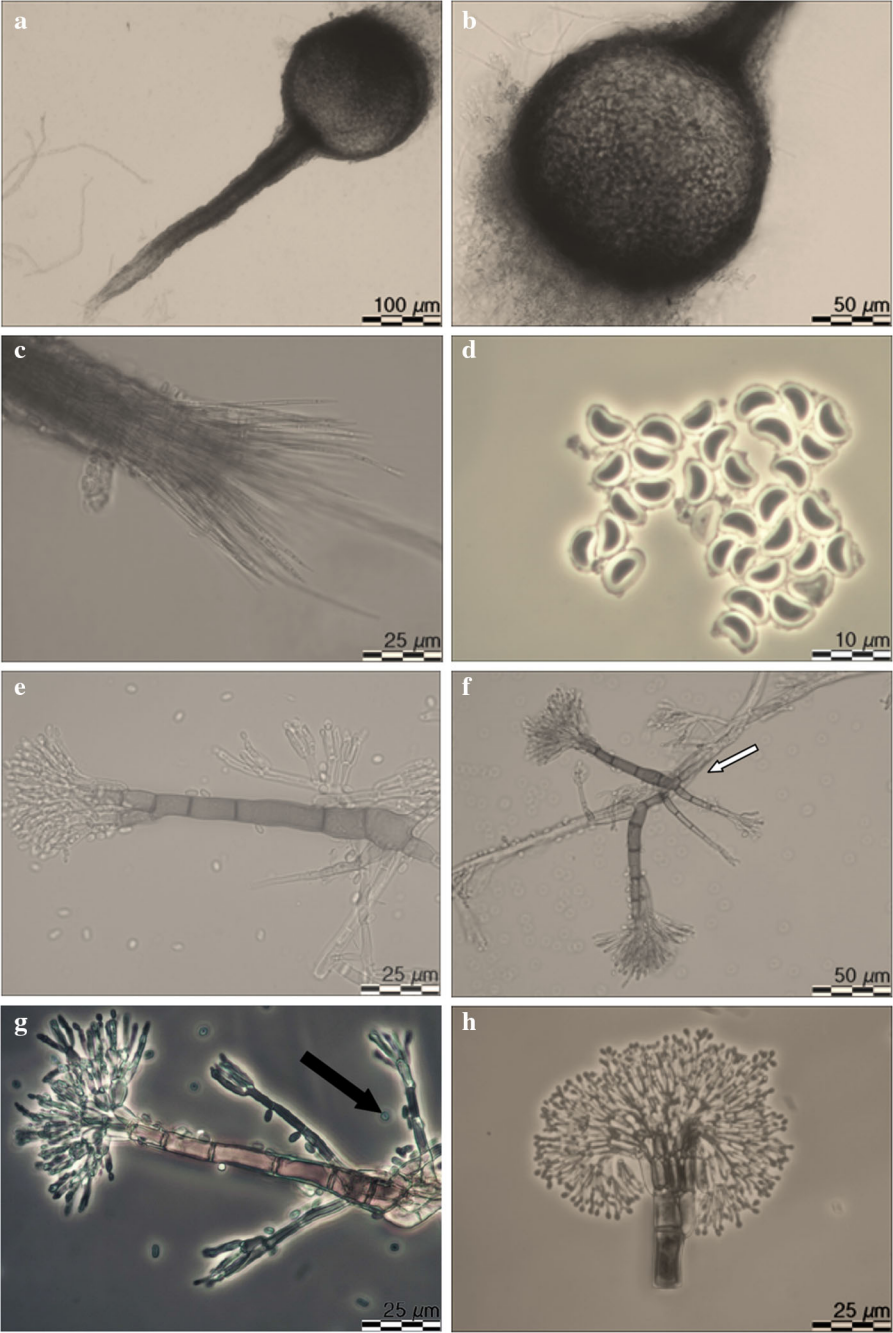

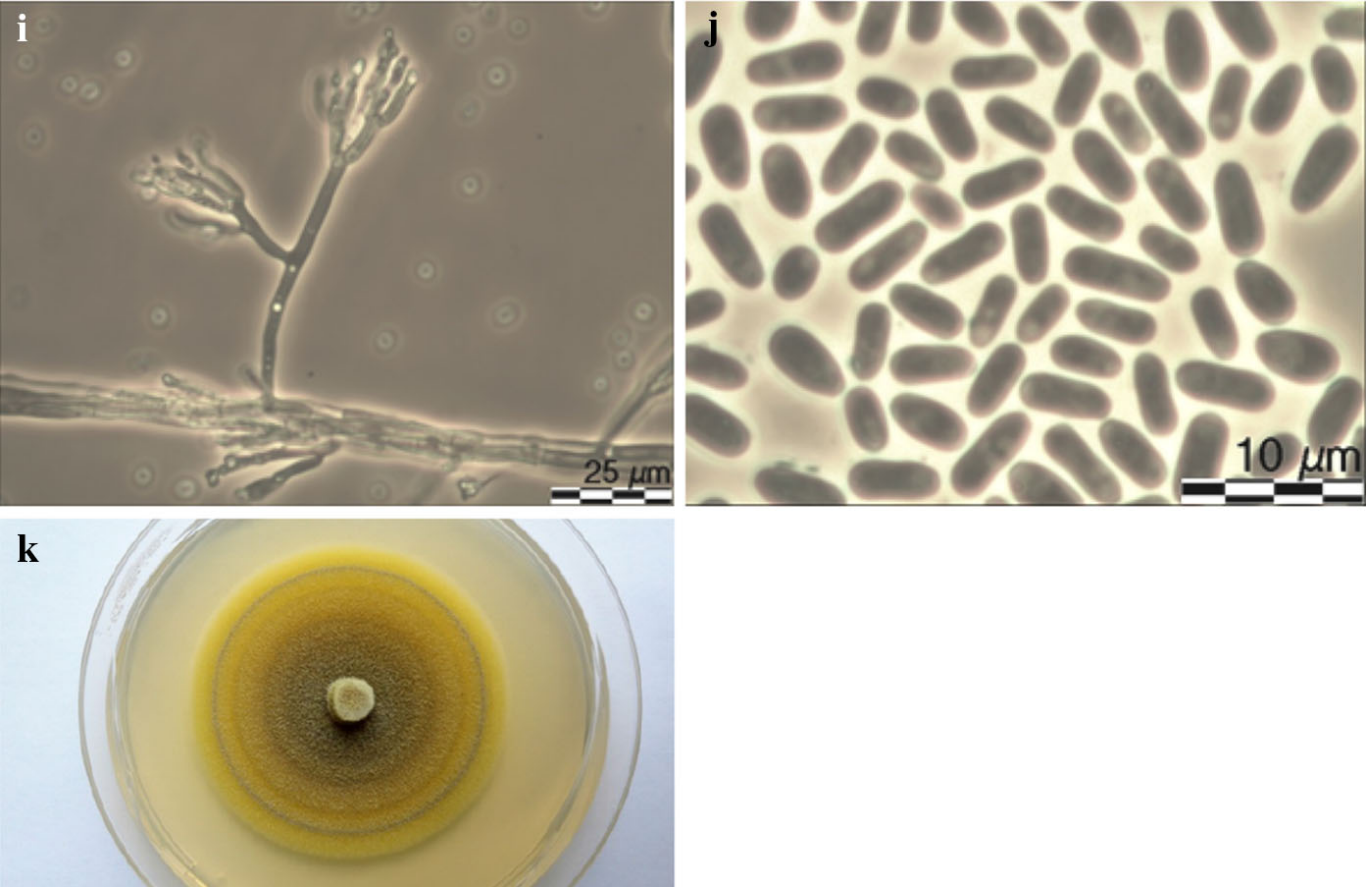
Morphological characteristics of *Leptographium flavum* sp. nov. (CBS 144099). **a** ascoma, **b** ascomatal base, **c** ostiolar hyphae, **d** ascospores, **e-g** conidiophores (type 1), black arrow indicates conidiophores of type 2, white arrow indicates secondary conidiophores emerging from base of primary conidiophores conidiophores (type 1),white arrow indicated secondary conidiophores emerging from base of primary conidiophores, **h** conidiogenous apparatus (conidiophores of type 1), **i** conidiophores (type 2), **j** conidia, **k** fourteen day old culture on MEA

*Leptographium flavum* R. Jankowiak and A. Ostafińska, sp. nov. (Fig. 11). MycoBank: 826761.

*Etymology* Name refers to yellow pigment produced by this fungus on malt agar.

*Sexual state* develops on malt agar in 21 days. *Perithecia* superficial on wood and agar, bases light brown to dark brown, globose, unornamented, 94.6–203.9 (mean 138.6 ± 29.1) μm in diameter, necks dark brown, cylindrical, straight, 285.2–377.2 (mean 336.6 ± 35) μm long (including ostiolar hyphae), 33–63.7 (mean 45.1 ± 8.4) μm wide at base, 18.9–35.9 (mean 27.2 ± 3) μm wide at the tip (Figs. 11a, b). *Ostiolar hyphae* present, pale brown, straight, septate, numerous, divergent, tapering at the tip, up to 84.6 μm long (Fig. 11c). *Asci* not seen. *Ascospores* one-celled, hyaline, orange section shaped in side view, ellipsoidal in face view, globose in end view, 4.4–6.4 (mean 5.4 ± 0.46) × 2.5–3.9 (mean 3.2 ± 0.37) μm including hyaline gelatinous sheath, 0.3–1.2 μm thick (Fig. 11d).

*Asexual states*: (1) *Conidiophores* (type 1): macronematous, arising directly from hyphae, without rhizoidal hyphae at the bases but often secondary conidiophores emerge from the base of the primary conidiophores, 111.6–189.1 (mean 143 ± 15.4) μm in length (Fig. 11e–g). *Stipes* erect, olivaceous, 3–8 septate, 69.3–124 (mean 91.2 ± 12.46) μm long from first basal septum to below primary branches, 4.1–8.4 (mean 6.1 ± 1) μm wide below primary branches, apical cell not swollen, and 6.8–10.4 (mean 8.5 ± 0.96) μm wide at base, basal cell not swollen. *Conidiogenous apparatus* 34.1–66.4 (mean 50.6 ± 7.5) μm long (excluding conidial mass) consisting of (1–) 2 (–3) series of branches-type B (more than two branches) (Jacobs and Wingfield 2001) (Fig. 11h). Primary branches dark olivaceous, cylindrical, smooth, 9.2–19.1 × 2.2–7.4 μm. *Conidiogenous cells* hyaline, tapering from base to apex, 17.7–43.1 (mean 28.3 ± 6.2) × 1.6–2.7 (mean 2.1 ± 0.27) μm. *Conidia* (Fig. 11j) hyaline, oblong to elliptical, sometimes obovate, 3.5–6 (mean 4.6 ± 0.62) × 1.2–3.3 (mean 1.9 ± 0.32) μm, accumulating around the conidiogenous apparatus in a hyaline mucilaginous mass.

(2): *Conidiophores* (type 2): occurring single or in groups, mostly on aerial mycelia but often emerge from the base of conidiophores as described above, macronematous, mononematous, 22.7–39.7 (mean 31.2 ± 4.2) μm in length including the conidial mass, rhizoid like structures absent (Figs. 11f, g, i). Stipes olivaceous, simple, 0–5 septate, 10.9–25.3 (mean 16.9 ± 2.7) μm long, 1.5–2.6 (mean 2 ± 0.3) μm wide below primary branches. Conidiogenous apparatus with 1–2 series of cylindrical branches. Primary branches, 2–4, light olivaceous, smooth, cylindrical aseptate, 6.8–26.7 (mean 11.5 ± 4.2) μm in length and 1.2–2.2 μm (mean 1.7 ± 0.3) wide, arrangement of the primary branches on the stipe-type B (more than two branches; Jacobs and Wingfield 2001). Conidiogenous cells discrete, 2–4 per branch, cylindrical, taper slightly at the apex, 6.7–13.7 (mean 9.3 ± 3.7) μm long and 1.0–2.3 (mean 1.5 ± 0.3) μm wide. *Conidia* hyaline, oblong to elliptical, 1.0–2.8 (mean 1.8 ± 0.44) × 0.7–2.0 (mean 1.2 ± 0.3) μm.

*Cultural characteristics* Colonies on MEA hyaline at first, later becoming vividly yellowish in the centre, concentric rings present, mycelium appressed and immersed (Fig. 11k). Yellow pigment was often produced on MEA. Colony margin smooth. Hyphae pale or light yellow in colour (Kornerup and Wanscher 1978), smooth, submerged in the medium and aerial mycelium abundant, not constricted at the septa, 0,9–4 (mean 2.2 ± 0.7) µm diam. Perithecia and asexual morphs co-occur in culture. Optimal growth temperature is 20 °C, radial growth rate 3.0 (± 0.1) mm/d, growth slightly reduced at 25 and 30 °C, no growth at 35 °C.

Host trees: *Quercus robur*, *Quercus rubra*
Substrate: natural fresh lesions (necrosis) on tree stem
Insect vector: *Anisandrus dispar*
Distribution: Poland

*Type material* POLAND, Wierzchosłwice, from *Quercus robur* wood, 7 October 2015, *R*. *Jankowiak*, holotype TUR http://mus.utu.fi/TFU.207257, culture ex-holotype CBS 144099 = CMW 51797; POLAND, Wierzchosłwice, from *Quercus robur* wood, 7 October 2015, *R*. *Jankowiak*, paratype TUR http://mus.utu.fi/TFU.207260, culture ex-paratype CBS 144102 = CMW 51800; POLAND, Resko, from *Aisandrus dispar* beetle infesting *Quercus robur*, 7 October 2016, *R. Jankowiak*, paratype TUR http://mus.utu.fi/TFU.207261, culture ex-paratype CBS 144105 = CMW 51804.

*Notes* The morphologically differences between *L. flavum* and *L. vulnerum* have been described above in section: Notes on *L. vulnerum*.

*Leptographium flavum* was found in 25 and 17% of *Q. rubra* and *Q. robur* lesions from trees, respectively, in six sample plots in southern Poland. It was also recorded on 2% of *A. dispar* beetles in one study plot in Poland.

## Discussion

Three new species of *Leptographium* were discovered from European hardwoods in this study. These new taxa are closely related to the species of the *G. olivacea* complex, but formed two separate, well-supported lineages. The first lineage consists of *L. tardum* sp. nov. and *O. brevicolle*, while in the second lineage resides *L. vulnerum* sp. nov. and *L. flavum* sp. nov. *G. francke*-*grosmanniae* has also affiliation to *L. vulnerum* and *L. flavum* based on analysis of the ITS and ß-tubulin sequences. The sexual states of these three new species were highly similar to those from species that comprise the *G. olivacea* complex (globose ascomata with cylindrical necks terminated by ostiolar hyphae at their apices, and orange-section shaped ascospores with cucullate gelatinous sheaths). However, in contrast to the species of the *G. olivacea* complex that produce synnematous asexual states, all three new species produce mononematous morphs. The three new species are characterized by dark pigmented mononematous conidiophores that resembling those produced by *G. francke*-*grosmanniae*, but with the difference that they did not form rhizoids. In addition, *L. vulnerum* and *L. flavum* produce a second type of mononematous conidiophores. These conidiophores have short, non-pigmented stipes developing directly from vegetative hyphae. Another important feature in distinguish these species is that these secondary conidiophores often develop from cells at the base of the primary conidiophores thus generating both type of conidiophores. These species can also be distinguished by their ecological niches; *L. tardum* has been found only in association with hardwoods-infesting insects while *L. vulnerum* and *L. flavum* were mainly associated with tree wounds.

The phylogenetic analysis and differences in asexual morphology of the three new species suggest that these species, together with *O. brevicolle* and *G. francke*-*grosmanniae* form two well-supported genetic lineages in *Leptographium s.l*. consisting only of hardwood-infecting species. *Ophiostoma brevicolle* was collected from *Populus tremuloides* Michx. in the USA (Davidson 1958), while *G. francke*-*grosmanniae* was isolated from the large timberworm beetle, *Hylecoetus dermestoides* (L.) (Coleoptera: Lymexylidae), infesting a *Quercus* sp. (Davidson 1971). In turn, *L. tardum* is a potential symbiotic associate of the European hardwood ambrosia beetle, *T. domesticum*, and the *T. signatum*. These ambrosia beetles attack different hardwood tree species in Europe and are known to prefer fallen or weakened trees and stumps (Wood and Bright 1992). *Leptographium tardum* has been isolated from *Trypodendron* spp. with variably frequencies, suggesting that the role of this fungus in the life-cycle of the ambrosia beetles is not important. The other newly described *Leptographium* species, *L. vulnerum* and *L. flavum* colonized fresh lesions on tree stems. Wounds and cracks that may be caused by animals, wind, frost, silvicultural practices and various arthropods (Heath et al. 2010), are essential entry points for ophiostomatoid fungi (Wingfield and Kemp 1993). However, despite the importance of ophiostomatoid fungi as possible serious tree pathogens, very little is known regarding the biodiversity of ophiostomatoid fungi associated with wounds on trees. Studies from South Africa, Australia and South America provide evidence that wounds on hardwood trees provide habitat for a large diversity of ophiostomatoid fungi (Barnes et al. 2003; Geldenhuis et al. 2004; Roux et al. 2004; Grobbelaar et al. 2010; Kamgan Nkuekam et al. 2008, 2011a, b, 2012; Osorio et al. 2016). Relatively little is known about ophiostomatoid species infecting wounds on hardwood trees in Europe. Recently, Kamgan Nkuekam et al. (2010) recorded *Ophiostoma quercus* (Georgev.) Nannf., *O. borealis*, G. Kamgan Nkuekam, H. Solheim and Z.W. de Beer and *O. denticiliatum* Linnak., Z.W. de Beer and M.J. Wingf. from wounds of native broad-leaved trees in Norway. Our studies have confirmed the ability of *Leptographium* spp. to infect hardwood trees in Europe although pathogenicity tests are needed to determine their pathogenicity towards hardwoods. In general, natural infections might be the result of wind or rain splash dispersal of propagules, or by transmission of inoculum by arthropod vectors (Malloch and Blackwell 1993; Six 2003; Klepzig and Six 2004; Hayslett et al. 2008; Juzwik et al. 2008). The association of *L. flavum* with *A. dispar*, as noted in this study, suggests that some beetle species, for example sap or ambrosia beetles that infest tree lesions can spread fungal spores in forest environments.

In comparison with conifer-infesting *Leptographium* spp., the taxonomy, diversity and pathogenicity of hardwood-infesting *Leptographium* species have been poorly studied. The results of this study have shown that *Leptographium* spp. are not rare inhabitants of hardwood forests in Europe, and suggest that these fungi may commonly infect wounds on hardwoods trees. Therefore, it will be important to expand these surveys to cover larger geographic areas including more habitats in Europe.

## Electronic supplementary material

Below is the link to the electronic supplementary material.

Supplementary material 1 (DOCX 75 kb)

## References

[CR1] Barnes I, Roux J, Wingfield BD, Dudzinski MJ, Old KM, Wingfield MJ (2003). *Ceratocystis pirilliformis*, a new species from *Eucalyptus nitens* in Australia. Mycologia.

[CR2] Darriba D, Taboada GL, Doallo R, Posada D (2012). jModelTest 2: more models, new heuristics and parallel computing. Nat Methods.

[CR3] Davidson RW (1958). Additional species of Ophiostomataceae from Colorado. Mycologia.

[CR4] Davidson RW (1971). New Species of Ceratocystis. Mycologia.

[CR5] De Beer ZW, Wingfield MJ, Seifert KA, De Beer ZW, Wingfield MJ (2013). Emerging lineages in the Ophiostomatales. The Ophiostomatoid fungi: expanding frontiers, CBS Biodiversity Series 12.

[CR6] Duong TA, De Beer ZW, Wingfield BD, Wingfield MJ (2012). Phylogeny and taxonomy of species in the *Grosmannia serpens* complex. Mycologia.

[CR7] Gardes M, Bruns TD (1993). ITS primers with enhanced specificity for Basidiomycetes—application to the identification of mycorrhiza and rusts. Mol Ecol.

[CR8] Geldenhuis MM, Roux J, Montenegro F, De Beer ZW, Wingfield MJ, Wingfield BD (2004). Identification and pathogenicity of *Graphium* and *Pesotum* species from machete wounds on *Schizolobium parahybum* in Ecuador. Fungal Divers.

[CR9] Glass NL, Donaldson GC (1995). Development of primer sets designed for use with the PCR to amplify conserved genes from filamentous ascomycetes. App Environ Microbiol.

[CR10] Goheen DJ, Cobb FW (1978). Occurrence of *Verticicladiella wageneri* and its perfect state, *Ceratocystis wageneri* sp. nov., in insect galleries. Phytopathology.

[CR11] Goidànich G (1936). II genre di Ascorniceti ‘*Grosmanni*’ G. Goid. Bolletino della Stazione di Patologia vegetale di Roma.

[CR12] Grobbelaar J, de Beer ZW, Bloomer P, Wingfield MJ, Wingfield BD (2010). *Ophiostoma tsotsi* sp. nov., a wound- infesting fungus of hardwood trees in Africa. Mycopathologia.

[CR13] Guindon S, Gascuel O (2003). A simple, fast and accurate method to estimate large phylogenies by maximum-likelihood. Syst Biol.

[CR14] Guindon S, Dufayard JF, Lefort V, Anisimova M, Hordijk W, Gascuel O (2010). New algorithms and methods to estimate maximum-likelihood phylogenies: assessing the performance of PhyML 3.0. Syst Biol.

[CR15] Hawksworth DL (2011). A new dawn for the naming of fungi: impacts of decisions made in Melbourne in July 2011 on the future publication and regulation of fungal names. IMA Fungus.

[CR16] Hayslett M, Juzwik J, Moltzan B (2008). Three *colopterus* beetle species carry the oak wilt fungus to fresh wounds on red oak in Missouri. Plant Dis.

[CR17] Heath RN, Linde M, Van Groeneveld H, Wingfield BD (2010). Factors influencing infection of *Acacia mearnsii* by the wilt pathogen *Ceratocystis albifundus* in South Africa. For Pathol.

[CR18] Jacobs K, Wingfield MJ (2001). *Leptographium* species: tree pathogens, insect associates, and agents of blue-stain.

[CR19] Jacobs K, Wingfield MJ, Wingfield BD (2001). Phylogenetic relationships in *Leptographium* based on morphological and molecular characters. Can J Bot.

[CR20] Jacobs K, Bergdahl DR, Wingfield MJ, Halik S, Seifert KA, Bright DE, Wingfield BD (2004). *Leptographium wingfieldii* introduced into North America and found associated with exotic *Tomicus piniperda* and native bark beetles. Mycol Res.

[CR21] Jacobs K, Eckhardt LG, Wingfield MJ (2006). *Leptographium profanum* sp. nov., a new species from hardwood roots in North America. Can J Bot.

[CR22] Jankowiak R, Strzałka B, Bilański P, Linnakoski R, Aas T, Solheim H, Groszek M, de Beer ZW (2017). Two new *Leptographium* spp. reveal an emerging complex of hardwood-infecting species in the Ophiostomatales. Antonie Van Leeuwenhoek.

[CR23] Juzwik J, Harrington TC, McDonald WL (2008). The origin of *Ceratocystis fagacearum*, the Oak wilt fungus. Annu Rev Phytopathol.

[CR24] Kamgan Nkuekam G, Jacobs K, De Beer ZW, Wingfield MJ, Roux J (2008). *Ceratocystis* and *Ophiostoma* species including three new taxa, associated with wounds on native South African trees. Fungal Divers.

[CR25] Kamgan Nkuekam G, Solheim H, De Beer ZW, Grobbelaar C, Jacobs K, Wingfield MJ, Roux J (2010). *Ophiostoma* species, including *Ophiostoma borealis* sp. nov., infecting wounds of native broad-leaved trees in Norway. Cryptogam Mycol.

[CR26] Kamgan Nkuekam G, De Beer ZW, Wingfield MJ, Mohammed C, Carnegie AJ, Pegg GS, Roux J (2011). *Ophiostoma* species (Ophiostomatales, Ascomycota), including two new taxa on eucalypts in Australia. Aust J Bot.

[CR27] Kamgan Nkuekam G, De Beer ZW, Wingfield MJ, Mohammed C, Carnegie AJ, Pegg GS, Roux J (2011). *Ophiostoma* species (Ophiostomatales, Ascomycota), including two new taxa on eucalypts in Australia. Aust J Bot.

[CR28] Kamgan Nkuekam G, Wingfield MJ, Mohammed C, Carnegie AJ, Pegg GS, Roux J (2012). *Ceratocystis* species, including two new species associated with nitidulid beetles, on eucalypts in Australia. Antonie Van Leeuwenhoek.

[CR29] Katoh K, Standley DM (2013). MAFFT multiple sequence alignment software version 7: improvements in performance and usability. Mol Biol Evo.

[CR30] Klepzig KD, Six DL (2004). Bark beetle-fungal symbiosis: context dependency in complex associations. Symbiosis.

[CR31] Kornerup A, Wanscher JH (1978). Methuen hanbook of colour.

[CR32] Lagerberg T, Lundberg G, Melin E (1927). Biological and practical researches into blueing in pine and spruce. Sver Skogsv För Tidskr.

[CR33] Lim YW, Massoumi Alamouti S, Kim JJ, Lee S, Breuil C (2004). Multigene phylogenies of *Ophiostoma clavigerum* and closely related species from bark beetle-attacked *Pinus* in North America. FEMS Microbiol Lett.

[CR34] Linnakoski R, De Beer ZW, Duong TA, Niemelä P, Pappinen A, Wingfield MJ (2012). *Grosmannia* and *Leptographium* spp. associated with conifer-infesting bark beetles in Finland and Russia including *Leptographium taigense* sp. nov. Antonie Van Leeuwenhoek.

[CR35] Malloch D, Blackwell M, Wingfield MJ, Seifert KA, Webber JF (1993). Dispersal biology of ophiostomatoid fungi. *Ceratocystis* and *Ophiostoma*: taxonomy, ecology, and pathology.

[CR36] Massoumi Alamouti S, Kim JJ, Humble LM, Uzunovic A, Breuil C (2007). Ophiostomatoid fungi associated with the northern spruce engraver, *Ips perturbatus*, in western Canada. Antonie Van Leeuwenhoek.

[CR37] Mathiesen A (1951). Einige neue *Ophiostoma*-arten in Schweden. Sv Bot Tidskr.

[CR38] O’Donnell K, Cigelnik E (1997). Two divergent intragenomic rDNA ITS2 types within a monophyletic lineage of the fungus *Fusarium* are nonorthologous. Mol Phylogent Evol.

[CR39] Okada G, Seifert KA, Takematsu A, Yamaoka Y, Miyazaki S, Tubaki K (1998). A molecular phylogenetic reappraisal of the *Graphium* complex based on 18S rDNA sequences. Can J Bot.

[CR40] Olchowecki A, Reid J (1974). Taxonomy of the genus *Ceratocystis* in Manitoba. Can J Bot.

[CR41] Osorio JA, De Beer ZW, Wingfield MJ, Roux J (2016). Ophiostomatoid fungi associated with mangroves in South Africa, including *Ophiostoma palustre* sp. nov. Antonie Van Leeuwenhoek.

[CR42] Paciura D, De Beer ZW, Jacobs K, Zhou XD, Ye H, Wingfield MJ (2010). Eight new *Leptographium* species associated with tree-infesting bark beetles in China. Persoonia.

[CR43] Rambaut A, Drummond AJ (2007) Tracer v1.4. http://beast.bio.ed.ac.uk/Tracer

[CR44] Robert V, Vu D, Amor AB (2013). MycoBank gearing up for new horizons. IMA Fungus.

[CR45] Ronquist F, Huelsenbeck JP (2003). MrBayes 3: Bayesian phylogenetic inference under mixed models. Bioinformatics.

[CR46] Roux J, VanWyk M, Hatting H, Wingfield MJ (2004). *Ceratocystis* species infecting stem wounds on *Eucalyptus grandis* in South Africa. Plant Pathol.

[CR47] Seifert KA, Wingfield MJ, Kendrick WB, Wingfield MJ, Seifert KA, Webber J (1993). A nomenclator for described species of *Ceratocystis*, *Ophiostoma*, *Ceratocystiopsis*, *Ceratostomella* and *Sphaeronaemella*. *Ceratocystis* and *Ophiostoma*: taxonomy, ecology and pathogenicity.

[CR48] Six DL, Bourtzis K, Miller T (2003). Bark beetle-fungus symbioses. Insect symbioses.

[CR49] Six DL, De Beer ZW, Duong TA, Carroll AL, Wingfield MJ (2011). Fungal associates of the lodgepole pine beetle, *Dendroctonus murrayanae*. Antonie Van Leeuwenhoek.

[CR50] Solheim H (1986). Species of Ophiostomataceae isolated from *Picea abies* infested by the bark beetle *Ips typographus*. Nord J Bot.

[CR51] Tamura K, Stecher G, Peterson D, Filipski A, Kumar S (2013). MEGA6: molecular evolutionary genetics analysis version 6.0. Mol Biol Evol.

[CR52] Taylor JW (2011). One Fungus = One Name: DNA and fungal nomenclature twenty years after PCR. IMA Fungus.

[CR53] Upadhyay HP (1981). A monograph of *Ceratocystis* and *Ceratocystiopsis*.

[CR54] White TJ, Bruns T, Lee S, Taylor J, Innis MA, Gelfand DH, Sninsky JJ, White TJ (1990). Amplification and direct sequencing of fungal ribosomal RNA genes for phylogenetics. PCR protocols: a guide to methods and applications.

[CR55] Wingfield MJ, Kemp GHJ, Van der Sidje HA (1993). Diseases of Pines, Eucalyptus and Wattles. South African forestry handbook.

[CR56] Wood SL, Bright DE (1992). A catalog of Scolytidae and Platypodiae (Coleoptera), Part 2. Taxonomic index. Great Basin Nat Mem.

[CR57] Wright EF, Cain RF (1961). New species of the genus *Ceratocystis*. Can J Bot.

[CR58] Yin M, Duong TA, Wingfield MJ, Zhou XD, De Beer ZW (2015). Taxonomy and phylogeny of the *Leptographium procerum* complex, including *L. sinense* sp. nov. and *L. longiconidiophorum* sp. nov. Antonie Van Leeuwenhoek.

[CR59] Zipfel RD, De Beer ZW, Jacobs K, Wingfield BD, Wingfield MJ (2006). Multi-gene phylogenies define *Ceratocystiopsis* and *Grosmannia* distinct from *Ophiostoma*. Stud Mycol.

