# Supplementary material for: Three new Leptographium spp. (Ophiostomatales) infecting hardwood trees in Norway and Poland
Source: Antonie Van Leeuwenhoek. 2018 Jul 6;111(12):2323–47. doi: 10.1007/s10482-018-1123-8 (PMC6245115; doi:10.1007/s10482-018-1123-8)
Supplement: Supplementary file 1 — Supplementary material 1 (DOCX 75 kb) [file 10482_2018_1123_MOESM1_ESM.docx]

**Table S1** Comparison of polymorphic sites of the β-tubulin gene for the three new taxa. Numbers written vertically above columns indicate the relative positions in the alignments.

| Species | Strains | *eeeeiiiiiiiiiiiiiiiiiiiiiiiiiiiiiieeeeeeeeeeee |
| --- | --- | --- |
|  |  | **11111111111111111222233**  **1234777778888888999999900000000011245678004500**  **8498567890123789012456902345678913795492062758** |
| **Taxon 1** | 2015-1552/3/10 | **TCTTTCTGCTCTAGACACGGTAACGCGAATCTACAGCTTCACAATG** |
|  | 2016**-**0625/1/1 | **TCTTTCTGCTCTAGACACGGTAACGCGAATCTACAGCTTCACAATG** |
|  | 2016-0637/1/2 | **TCTTTCTGCTCTAGACACGGTAACGCGGATCTACAGCTTCACAATG** |
|  | 2016-0676/2/2 | **TCTTTCTGCTCTAGACACGGTAACGCGGATCTACAGCTTCACAATG** |
|  | 2016**-**1614**/3/1** | **TCTTTCTGCTCTAGACACGGTAACGCGGATCTACAGCTTTACAATG** |
|  | 2016-1627/2/1 | **TCTTTCTGCTCTAGACACGGTAACGCGGATCTACAGCTTTACAATG** |
|  | 2016-1631/1/2 | **TCTTTCTGCTCTAGACACGGTAACGCGGATCTACAGCTTCACAATG** |
|  | KFL118TD | **CTTTTCTGCTCTAGACACGGTAACGCGGATCTACAGCTTCACAATG** |
|  | KFL29715TD | **CTTTTCTGCTCTAGACACGGTAACGCGGATCTACAGCTTCACAATG** |
|  | KFL5814TD | **CTTTTCTGCTCTAGTCACGGTAACGCGAATCTACAGCTTCACAATG** |
|  | KFL6014TD | **CTTTTCTGCTCTAGTCACGGTAACGCGAATCTACAGCTTCACAATG** |
| **Taxon 2** | KFL110016NDBCZ | **TCCCGTATTCTATCCTTGTTACTACGATGCTATTCGTGGTCCTCCC** |
|  | KFL111416NDB | **TCCCGTATTCTATCCTTGTTACTACGATGCTATTCGTGGTCCTCCC** |
|  | KFL27216NBK | **TCCCGTATTCTATCCTTGTTACTACGATGCTATTCGTGGTCCTCCC** |
|  | KFL27316NGB | **TCCCGTATTCTATCCTTGTTACTACGATGCTATTCGTGGTCCTCCC** |
|  | KFL27716NGB | **TCCCGTATTCTATCCTTGTTACTACGATGCTATTCGTGGTCCTCCC** |
| **Taxon 3** | KFL103416XD | **TCCCGTATTCTATCTTTGTTATTACGATGCTATTCATGGTCTTCCC** |
|  | KFL104316XD | **TCCCGTATTCTATCTTTGTTATTACGATGCTATTCATGGTCTTCCC** |
|  | KFL111316NDB | **TCCCGTATTCTATCTTTGTTATTACGATGCTATTCATGGTCTTCCC** |
|  | KFL111916NDB | **TCCCGTATTCTATCTTTGTTATTACGATGCTATTCATGGTCTTCCC** |
|  | KFL114416NDB | **TCCCGTATTCTATCTTTGTTATTACGATGCTATTCATGGTCTTCCC** |
|  | KFL114916NDB | **TCCCGTATTCTATCTTTGTTATTACGATGCTATTCATGGTCTTCCC** |
|  | KFL1315NDB | **TCCCGTATTCTATCTTTGTTATTACGATGCTATTCATGGTCTTCCC** |
|  | KFL14516NDBCZ | **TCCCGTATTCTATCTTTGTTATTACGATGCTATTCATGGTCTTCCC** |
|  | KFL24516ND | **TCCCGTATTCTATCTTTGTTATTACGATGCTATTCATGGTCTTCCC** |
|  | KFL41716NDB | **TCCCGTATTCTATCTTTGTTATTACGATGCTATTCATGGTCTTCCC** |
|  | KFL42016NDB | **TCCCGTATTCTATCTTTGTTATTACGATGCTATTCATGGTCTTCCC** |
|  | KFL615NDB | **TCCCGTATTCTATCTTTGTTATTACGATGCTATTCATGGTCTTCCC** |

* “i” indicates introns, while “e” represents exons in the gene

**Table S2** Comparison of polymorphic sites of the translation elongation factor 1-α (TEF 1-α) gene for the three new taxa. Numbers written vertically above columns indicate the relative positions in the alignments.

| Species | Strains | ***iiiiiiiiiiiiiiiiiiiiiiiiiiiiiiiiiiiiiiiiiiiiiiiiiiiiiiiiiiiiiiiiiiiiiiiiiiiiiiiiiiiiiiiiiiiiiiiiiiiiiiiiiiiiiiiiiiiiiiiiiiiiiiiiiiiiiiiieeeeeeiiiiiiiiiiiiiiiiiiiiiiiieeeeee** |
| --- | --- | --- |
|  |  | **111111111111111111111111111111111111111111111111111111111111112222222222222222222222222333333333333333333333333333333333444455555555555555555555555555566778**  **5555666777888999000001111112222222222333333334444445555566777777778888899999990111123334455566677777899000000023333444555555666666777889127911444444455555556666778888967143**  **1235346167356018167890246890123456789012346791346890168989012345890135612345679023444560834557812456109345678992368145235689012689167592029434012568901347890124384569687999** |
| **Taxon 1** | 2015-1552/3/10 | **TCCCTACG-TA----TA---CCTCTCCAGGCG--------AAAACGCGGATTTTGC--------T---CGG---------AACCTTTTCCG-AAACGAACTTG-------CCGCGC---TCATCACTG-GACCGCACTTCAACTATGATTAACTGTTGAATTACCTCTTTCC** |
|  | 2016**-**0625/1/1 | **TCCCTACG-TA----TA---CCTCTCCAGGCG--------AAAACGCGGATTTTGC--------T---CGG---------AACCTTTTCCG-AAACGAACTTG-------CCGCGC---TCAATACTG-GACCGCACTTCAACTATGATTAACTGTTGAGTTACCTCTTTCC** |
|  | 2016-0637/1/2 | **TCCCTACG-TA----TA---CCTCTCCAGGCG--------AAAACGCGGATTTTGC--------T---CGG---------AACCTTTTCCG-AAACGAACTTG-------CCGCGC---TCATCACTG-GACCGCACTTCAACTATGATTAACTGTTGAATTACCTCTTTCC** |
|  | 2016-0676/2/2 | **TCCCTACG-TA----TA---CCTCTCCAGGCG--------AAAACGCGGATTTTGC--------T---CGG---------AACCTTTTCCG-AAACGAACTTG-------CCGCGC---TCAATACTG-GACCGCACTTCAACTATGATTAACTGTTGAGTTACCTCTTTCC** |
|  | 2016**-**1614**/3/1** | **TCCCTACG-TA----TA---CCTCTCCAGGCG--------AAAACGCGGATTTTGC--------T---CGG---------AACCTTTTCCG-AAACGAACTTG-------CCGCGC---TCAATACTG-GACCGCACTTCAACTATGATTAACTGTTGAGTTACCTCTTTCC** |
|  | 2016-1627/2/1 | **TCCCTACG-TA----TA---CCTCTCCAGGCG--------AAAACGCGGATTTTGC--------T---CGG---------AACCTTTTCCG-AAACGAACTTG-------CCGCGC---TCAATACTG-GACCGCACTTCAACTATGATTAACTGTTGAGTTACCTCTTTCC** |
|  | 2016-1631/1/2 | **TCCCTACG-TA----TA---CCTCTCCAGGCG--------AAAACGCGGATTTTGC--------T---CGG---------AACCTTTTCCG-AAACGAACTTG-------CCGCGC---TCAATACTG-GACCGCACTTCAACTATGATTAACTGTTGAGTTACCTCTTTCC** |
|  | KFL118TD | **TCCCTACG-TA----TA---CCTCTCCAGGCG--------AAAACGCGGATTTTGC--------T---CGG---------AACCTTTTCCG-AAACGAACTTG-------CCGCGC---TCAATACTG-GACCGCACTTCAACTATGATTAACTGTTGAGTTACCTCTTTCC** |
|  | KFL29715TD | **TCCCTACG-TA----TA---CCTCTCCAGGCG--------AAAACGCGGATTTTGC--------T---CGG---------AACCTTTTCCG-AAACGAACTTG-------CCGCGC---TCATCACTG-GACCGCACTTCAACTATGATTAACTGTTGAATTACCTCTTTCC** |
|  | KFL5814TD | **TCCCTACG-TA----TA---CCTCTCCAGGCG--------AAAACGCGGATTTTGC--------T---CGG---------AACCTTTACCG-AAATGAACTTG-------CCGCGC---TCAATACTG-GACCGCACTTCAACTATGATTAACTGTTGAGTTACCTCTTTCC** |
|  | KFL6014TD | **TCCCTACG-TA----TA---CCTCTCCAGGCG--------AAAACGCGGATTTTGC--------T---CGG---------AACCTTTACCG-AAATGAACTTG-------CCGCGC---TCAATACTG-GACCGCACTTCAACTATGATTAACTGTTGAGTTACCTCTTTCC** |
| **Taxon 2** | KFL110016NDBCZ | **CTTA-GTCGCCGCATG-CCTTTACAAAGATTCTCAAGTCCCAGCGAAAATCAAAATTCTTAGCCGGCATCACCTCTGGTTTCATACGTATTTGGC--GGGCCATGGACTGTGCTGTTCACTCATCTGGCTGT-AGTGGCGCGTC-GCT-GTTGC---ACCCGCCACTCCCTG** |
|  | KFL111416NDB | **CTTA-GTCGCCGCATG-CCTTTACAAAGATTCTCAAGTCCCAGCGAAAATCAAAATTCTTAGCCGGCATCACCTCTGGTTTCATACGTATTTGGC--GGGCCATGGACTGTGCTGTTCACTCATCTGGCTGT-AGTGGCGCGTC-GCT-GTTGC---ACCCGCCACTCCCTG** |
|  | KFL27216NBK | **CTTA-GTCGCCGCATG-CCTTTACAAAGATTCTCAAGTCCCAGCGAAAATCAAAATTCTTAGCCGGCATCACCTCTGGTTTCATACGTATTTGGC--GGGCCATGGACTGTGCTGTTCACTCATCTGGCTGT-AGTGGCGCGTC-GCT-GTTGC---ACCCGCCACTCCCTG** |
|  | KFL27316NGB | **CTTA-GTCGCCGCATG-CCTTTACAAAGATTCTCAAGTCCCAGCGAAAATCAAAATTCTTAGCCGGCATCACCTCTGGTTTCATACGTATTTGGC--GGGCCATGGACTGTGCTGTTCACTCATCTGGCTGT-AGTGGCGCGTC-GCT-GTTGC---ACCCGCCACTCCCTG** |
|  | KFL27716NGB | **CTTA-GTCGCCGCATG-CCTTTACAAAGATTCTCAAGTCCCAGCGAAAATCAAAATTCTTAGCCGGCATCACCTCTGGTTTCATACGTATTTGGC--GGGCCATGGACTGTGCTGTTCACTCATCTGGCTGT-AGTGGCGCGTC-GCT-GTTGC---ACCCGCCACTCCCTG** |
| **Taxon 3** | KFL103416XD | **CT-ATGTCGCCGCATTGCCTTCATAAAAGTTC-------CCGGCGAAAATCCTAGT-CTTAGCCGGCATCACCTCTGGTTTCATACGTATTTGGC--AAGCTATGCACTGTGCCATTCACTCATCCGACTGT-AGTGGCGCGTCCGCT-GTTGC---ACCCGCTACCCCCTG** |
|  | KFL104316XD | **CT-ATGTCGCCGCATTGCCTTCATAAAAGTTC-------CCGGCGAAAATCCTAGT-CTTAGCCGGCATCACCTCTGGTTTCATACGTATTTGGC--AAGCTATGCACTGTGCCATTCACTCATCCGACTGT-AGTGGCGCGTCCGCT-GTTGC---ACCCGCTACCCCCTG** |
|  | KFL111316NDB | **CT-ATGTCGCCGCATTGCCTTCATAAAAGTTC-------CCGGCGAAAATCCTAGT-CTTAGCCGGCATCACCTCTGGTTTCATACGTATTTGGC--AAGCTATGCACTGTGCCATTCACTCATCCGACTGT-AGTGGCGCGTCCGCT-GTTGC---ACCCGCTACCCCCTG** |
|  | KFL111916NDB | **CT-ATGTCGCCGCATTGCCTTCATAAAAGTTC-------CCGGCGAAAATCCTAGT-CTTAGCCGGCATCACCTCTGGTTTCATACGTATTTGGC--AAGCTATGCACTGTGCCATTCACTCATCCGACTGT-AGTGGCGCGTCCGCT-GTTGC---ACCCGCTACCCCCTG** |
|  | KFL114416NDB | **CT-ATGTCGCCGCATTGCCTTCATAAAAGTTC-------CCGGCGAAAATCCTAGT-CTTAGCCGGCATCACCTCTGGTTTCATACGTATTTGGC--AAGCTATGCACTGTGCCATTCACTCATCCGACTGT-AGTGGCGCGTCCGCT-GTTGC---ACCCGCTACCCCCTG** |
|  | KFL114916NDB | **CT-ATGTCGCCGCATTGCCTTCATAAAAGTTC-------CCGGCGAAAATCCTAGT-CTTAGCCGGCATCACCTCTGGTTTCATACGTATTTGGC--AAGCTATGCACTGTGCCATTCACTCATCCGACTGT-AGTGGCGCGTCCGCT-GTTGC---ACCCGCTACCCCCTG** |
|  | KFL1315NDB | **CT-ATGTCGCCGCATTGCCTTCATAAAAGTTC-------CCGGCGAAAATCCTAGT-CTTAGCCGGCATCACCTCTGGTTTCATACGTATTTGGC--AAGCTATGCACTGTGCCATTCACTCATCCGACTGT-AGTGGCGCGTCCGCT-GTTGC---ACCCGCTACCCCCTG** |
|  | KFL14516NDBCZ | **CT-ATGTCGCCGCATTGCCTTCATAAAAGTTC-------CCGGCGAAAATCCTAGT-CTTAGCCGGCATCACCTCTGGTTTCATACGTATTTGGC--AAGCTATGCACTGTGCCATTCACTCATCCGACTGT-AGTGGCGCGTCCGCT-GTTGC---ACCCGCTACCCCCTG** |
|  | KFL24516ND | **CT-ATGTCGCCGCATTGCCTTCATAAAAGTTC-------CCGGCGAAAATCCTAGT-CTTAGCCGGCATCACCTCTGGTTTCATACGTATTTGGC--AAGCTATGCACTGTGCCATTCACTCATCCGACTGT-AGTGGCGCGTCCGCT-GTTGC---ACCCGCTACCCCCTG** |
|  | KFL41716NDB | **CT-ATGTCGCCGCATTGCCTTCATAAAAGTTC-------CCGGCGAAAATCCTAGT-CTTAGCCGGCATCACCTCTGGTTTCATACGTATTTGGC--AAGCTATGCACTGTGCCATTCACTCATCCGACTGT-AGTGGCGCGTCCGCT-GTTGC---ACCCGCTACCCCCTG** |
|  | KFL42016NDB | **CT-ATGTCGCCGCATTGCCTTCATAAAAGTTC-------CCGGCGAAAATCCTAGT-CTTAGCCGGCATCACCTCTGGTTTCATACGTATTTGGC--AAGCTATGCACTGTGCCATTCACTCATCCGACTGT-AGTGGCGCGTCCGCT-GTTGC---ACCCGCTACCCCCTG** |
|  | KFL615NDB | **CT-ATGTCGCCGCATTGCCTTCATAAAAGTTC-------CCGGCGAAAATCCTAGT-CTTAGCCGGCATCACCTCTGGTTTCATACGTATTTGGC--AAGCTATGCACTGTGCCATTCACTCATCCGACTGT-AGTGGCGCGTCCGCT-GTTGC---ACCCGCTACCCCCTG** |

* “i” indicates introns, while “e” represents exons in the gene

**Table S3** Comparison of polymorphic sites of the ACT gene for the three new taxa. Numbers written vertically above columns indicate the relative positions in the alignments.

| Species | Strains | *eeeeeeeeeeeeeeeeeeeeeeeeeeeeeiiiiiiiiiiiiiiiiiiiiiiiiiiiiiiieeeee |
| --- | --- | --- |
|  |  | 1111222222233344455555666677777777777777777777777777777777888  1251679012245712312804679157900000000111111122222233334444578011  87369431924727513892538650432601456789012345712347912680379170703 |
| **Taxon 1** | 2015-1552/3/10 | **CTTTGGGCTTCGTTGCCCCGAGCCTCCCCGAC------------GAAGCGAGGGAGTGCCGTTCG** |
|  | 2016**-**0625/1/1 | **CTTTGGGCTTCGTTGCCCCGAGCCTCCCCGAC------------GAAGCGAGGGAGTGCCGTTCG** |
|  | 2016-0637/1/2 | **CTTTGGGCTTCGTTGCCCCGAGCCTCCCCGAC------------GAAGCGAGGGAGTGCCGTTCG** |
|  | 2016-0676/2/2 | **CTTTGGGCTTCGTTGCCCCGAGCCTCCCCGAC------------GAAGCGAGGGAGTGCCGTTCG** |
|  | 2016**-**1614**/3/1** | **CTTTGGGCTTCGTTGCCCCGAGCCTCCCCGAC------------GAAGCGAGGGAGTGCCGTTCG** |
|  | 2016-1627/2/1 | **CTTTGGGCTTCGTTGCCCCGAGCCTCCCCGAC------------GAAGCGAGGGAGTGCCGTTCG** |
|  | 2016-1631/1/2 | **CTTTGGGCTTCGTTGCCCCGAGCCTCCCCGAC------------GAAGCGAGGGAGAGCCGTTCG** |
|  | KFL118TD | **CTTTGGGCTTCGTTGCCCCGAGCCTCCCCGAC------------GAAGCGAGGGAGTGCCGTTCG** |
|  | KFL29715TD | **CTTTGGGCTTCGTTGCCCCGAGCCTCCCCGAC------------GAATTGAGGGAATGCCGTTCG** |
|  | KFL5814TD | **CTTTGGGCTTCGTTGCCCCGAGCCTCCCCGAC------------GAAGCGAGGGAGTGCCGTTCG** |
|  | KFL6014TD | **CTTTGGGCTTCGTTGCCCCGAGCCTCCCCGAC------------GAAGCGAGGGAGTGCCGTTCG** |
| **Taxon 2** | KFL110016NDBCZ | **TTCCCCGTCCCCGCGGGTTCGGCCGGTTTTGGGGAAGCGGACGCACGGATGAAACATCTTCCCTT** |
|  | KFL111416NDB | **TTCCCCGTCCCCGCGGGTTCGGCCGGTTTTGGGGAAGCGGACGCACGGATGAAACATCTTCCCTT** |
|  | KFL27216NBK | **TTCCCCGTCCCCGCGGGTTCGGCCGGTTTTGGGGAAGCGGACGCACGGATGAAACATCTTCCCTT** |
|  | KFL27316NGB | **TTCCCCGTCCCCGCGGGTTCGGCCGGTTTTGGGGAAGCGGACGCACGGATGAAACATCTTCCCTT** |
|  | KFL27716NGB | **TTCCCCGTCCCCGCGGGTTCGGCCGGTTTTGGGGAAGCGGACGCACGGATGAAACATCTTCCCTT** |
| **Taxon 3** | KFL103416XD | **TCCCCCATCCTCGCAGGTTCGATTAGTTCTGCGGAAGTGGACGCACGGACGAAACATCTCCCCTT** |
|  | KFL104316XD | **TCCCCCATCCTCGCAGGTTCGATTAGTTCTGCGGAAGTGGACGCACGGACGAAACATCTCCCCTT** |
|  | KFL111316NDB | **TCCCCCATCCTCGCAGGTTCGATTAGTTCTGCGGAAGCGGACGCACGGACGAAACATCTCCCCTT** |
|  | KFL111916NDB | **TCCCCCATCCTCGCAGGTTCGATTAGTTCTGCGGAAGCGGACGCACGGACGAAACATCTCCCCTT** |
|  | KFL114416NDB | **TCCCCCATCCTCGCAGGTTCGATTAGTTCTGCGGAAGTGGACGCACGGACGAAACATCTCCCCTT** |
|  | KFL114916NDB | **TCCCCCATCCTCGCAGGTTCGATTAGTTCTGCGGAAGTGGACGCACGGACGAAACATCTCCCCTT** |
|  | KFL1315NDB | **TCCCCCATCCTCGCAGGTTCGATTAGTTCTGCGGAAGCGGACGCACGGACGAAACATCTCCCCTT** |
|  | KFL14516NDBCZ | **TCCCCCATCCTCGCAGGTTCGATTAGTTCTGCGGAAGCGGACGCACGGACGAAACATCTCCCCTT** |
|  | KFL24516ND | **TCCCCCATCCTCGCAGGTTCGATTAGTTCTGCGGAAGTGGACGCACGGACGAAACATCTCCCCTT** |
|  | KFL41716NDB | **TCCCCCATCCTCGCAGGTTCGATTAGTTCTGCGGAAGTGGACGCACGGACGAAACATCTCCCCTT** |
|  | KFL42016NDB | **TCCCCCATCCTCGCAGGTTCGATTAGTTCTGCGGAAGTGGACGCACGGACGAAACATCTCCCCTT** |
|  | KFL615NDB | **TCCCCCATCCTCGCAGGTTCGATTAGTTCTGCGGAAGCGGACGCACGGACGAAACATCTCCCCTT** |

* “i” indicates introns, while “e” represents exons in the gene

**Table S4** Comparison of polymorphic sites of the CAL gene for the three new taxa. Numbers written vertically above columns indicate the relative positions in the alignments.

| Species | Strains | *iiiiiiiiiiiiiiiiiiiieeeeeeeiiiiiiiiiiiiiiiiiiiiiiiiiiiiiiiiiiiiieeeeeeeeeeeeeiiiiiiiiiiiiiiiiiiiiiiiiiiiii |
| --- | --- | --- |
|  |  | 1111111111122222222222222222222222222222233333344444444444444444444555555555555555  111222222333344445577890179999999900000000011111111112222333444606789900124577888899999999000000111111112  8679012357235901241214984390123456802345678901234567892357018578196576938137929245602345679124789012345671 |
| **Taxon 1** | 2015-1552/3/10 | **TCTA----GCAATTGCAATTTATACGTG------TGAA-----------GTGTAGGGCAACGTGTATCCCGCTTTGCAGTACAACCAGACAAGATAGCAGAGAGCG** |
|  | 2016-0625/1/1 | **TCTA----GCAATTGCAATTTATACGTG------TGAA-----------GTGTAGGGCAACGTGTATCCCGCTTTGCAGTACAACCAGACAAGATAGCAGAGAGCG** |
|  | 2016-0676/2/2 | **TCTA----GCAATTGCAATTTATACGTG------TGAA-----------GTGTAGGGCAACGTGTATCCCGCTTTGCAGTACAACCAGACAAGATAGCAGAGAGCG** |
|  | 2016-1614/3/1 | **TCTA----GCAATTGCAATTTATACGTG------TGAA-----------GTGTAGGGCAACGTGTATCCCGCTTTGCAGTACAACCAGACAAGATAGCAGAGAGCG** |
|  | 2016-1631/1/2 | **TCTA----GCAATTGCAATTTATACGTG------TGAA-----------GTGTAGGGCAACGTGTATCCCGCTTTGCAGTACAACCAGACAAGATAGCAGAGAGCG** |
|  | KFL118TD | **TCTA----GCAATTGCAATTTATACGTG------TGAA-----------GTGTAGGGCAACGTGTATCCCGCTTTGCAGTACAACCAGACAAGATAGCAGAGAGCG** |
|  | KFL29715TD | **TCTA----GCAATTGCAATTTATACGTG------TGAA-----------GTGTAGGGCAACGTGTATCCCGCTTTGCAGTACAACCAGACAAGATAGCAGAGAGCG** |
|  | KFL5814TD | **TCTA----GCAATTGCAATTTATACGTG------TGAA-----------GTGTAGGGCAACGTGTATCCCGCTTTGCAGTACAACCAGACAAGATAGCAGAGAGCG** |
|  | KFL6014TD | **TCTA----GCAATTGCAATTTATACGTG------TGAA-----------GTGTAGGGCAACGTGTATCCCGCTTTGCAGTACAACCAGACAAGATAGCAGAGAGCG** |
| **Taxon 2** | KFL110016NDBCZ | **CCCACTGCAGCGCCACGGGGCGCGAACA------CGGGGGTGTGTGTGTGTGTGAAACGGTCAACGCCGCGCGCCGCGAATTTCTT--TTGGAC-------AGATA** |
|  | KFL111416NDB | **CCCACTGCAGCGCCACGGGGCGCGAACA------CGGGGGTGTGTGTGTGTGTGAAACGGTCAACGCCGCGCGCCGCGAATTTCTT--TTGGAC-------AGATA** |
|  | KFL27216NBK | **CCCACTGCAGCGCCACGGGGCGCGAACA------CGGGGGTGTGTGTGTGTGTGAAACGGTCAACGCCGCGCGCCGCGAATTTCTT--TTGGAC-------AGATA** |
|  | KFL27316NGB | **CCCACTGCAGCGCCACGGGGCGCGAACA------CGGGGGTGTGTGTGTGTGTGAAACGGTCAACGCCGCGCGCCGCGAATTTCTT--TTGGAC-------AGATA** |
|  | KFL27716NGB | **CCCACTGCAGCGCCACGGGGCGCGAACA------CGGGGGTGTGTGTGTGTGTGAAACGGTCAACGCCGCGCGCCGCGAATTTCTT--TTGGAC-------AGATA** |
| **Taxon 3** | KFL103416XD | **CACGCTGCAGCGCCATGGGGCGCGAACAGTGTGTTTTGTGTGTGTG--------AAATGGTGAACGCTGCATGCCATGAATTTCTT--TTGGAC-------AGATA** |
|  | KFL104316XD | **CACGCTGCAGCGCCATGGGGCGCGAACAGTGTGTTTTGTGTGTGTG--------AAATGGTGAACGCTGTATGCCATGAATTTCTT--TTGGAC-------AGATA** |
|  | KFL111316NDB | **CACGCTGCAGTGCCATGGGGCGCGAACAGTGTGTTTTGTGTGTGTGTGTGT--GAAATGGTGAACGCTGCATGCCATGAATTTCTT--TTGGAC-------AGATA** |
|  | KFL111916NDB | **CACGCTGCAGCGCCATGGGGCGCGAACAGTGTGTTTTGTGTGTGTG--------AAATGGTGAACGCTGTATGCCATGAATTTCTT--TTGGAC-------AGATA** |
|  | KFL114416NDB | **CACGCTGCAGTGCCATGGGGCGCGAACAGTGTGTTTTGTGTGTGTGTGTGTGTGAAATGGTGAACGCTGTATGCCATGAATTTCTT--TTGGAC-------AGATA** |
|  | KFL114916NDB | **CACGCTGCAGTGCCATGGGGCGCGAACAGTGTGTTTTGTGTGTGTGTGTGTGTGAAATGGTGAACGCTGTATGCCATGAATTTCTT--TTGGAC-------AGATA** |
|  | KFL1315NDB | **CACGCTGCAGCGCCATGGGGCGCGAACAGTGTGTTTTGTGTGTGTG--------AAATGGTGAACGCTGTATGCCATGAATTTCTT--TTGGAC-------AGATA** |
|  | KFL14516NDBCZ | **CACGCTGCAGTGCCATGGGGCGCGAACAGTGTGTTTTGTGTGTGTGTGTGT--GAAATGGTGAACGCTGTATGCCATGAATTTCTT--TTGGAC-------AGATA** |
|  | KFL24516ND | **CACGCTGCAGCGCCATGGGGCGCGAACAGTGTGTTTTGTGTGTGTG--------AAATGGTGAACGCTGCATGCCATGAATTTCTT--TTGGAC-------AGATA** |
|  | KFL41716NDB | **CACGCTGCAGTGCCATGGGGCGCGAACAGTGTGTTTTGTGTGTGTGTGTGTGTGAAATGGTGAACGCTGTATGCCATGAATTTCTT--TTGGAC-------AGATA** |
|  | KFL42016NDB | **CACGCTGCAGTGCCATGGGGCGCGAACAGTGTGTTTTGTGTGTGTGTGTGTGTGAAATGGTGAACGCTGTATGCCATGAATTTCTT--TTGGAC-------AGATA** |
|  | KFL615NDB | **CACGCTGCAGTGCCATGGGGCGCGAACAGTGTGTTTTGTGTGTGTGTGTGTGTGAAATGGTGAACGCTGTATGCCATGAATTTCTT--TTGGAC-------AGATA** |

* “i” indicates introns, while “e” represents exons in the gene
